# Low-dose proton radiation effects in a transgenic mouse model of Alzheimer’s disease – Implications for space travel

**DOI:** 10.1371/journal.pone.0186168

**Published:** 2017-11-29

**Authors:** Emil Rudobeck, John A. Bellone, Attila Szücs, Kristine Bonnick, Shalini Mehrotra-Carter, Jerome Badaut, Gregory A. Nelson, Richard E. Hartman, Roman Vlkolinský

**Affiliations:** 1 Department of Basic Sciences, School of Medicine, Loma Linda University, Loma Linda, CA, United States of America; 2 Department of Psychology, School of Behavioral Health, Loma Linda University, Loma Linda, CA, United States of America; 3 BioCircuits Institute, University of California San Diego, La Jolla, CA, United States of America; 4 Department of Physiology, School of Medicine, Loma Linda University, Loma Linda, CA, United States of America; ENEA Centro Ricerche Casaccia, ITALY

## Abstract

Space radiation represents a significant health risk for astronauts. Ground-based animal studies indicate that space radiation affects neuronal functions such as excitability, synaptic transmission, and plasticity, and it may accelerate the onset of Alzheimer’s disease (AD). Although protons represent the main constituent in the space radiation spectrum, their effects on AD-related pathology have not been tested. We irradiated 3 month-old APP/PSEN1 transgenic (TG) and wild type (WT) mice with protons (150 MeV; 0.1–1.0 Gy; whole body) and evaluated functional and biochemical hallmarks of AD. We performed behavioral tests in the water maze (WM) before irradiation and in the WM and Barnes maze at 3 and 6 months post-irradiation to evaluate spatial learning and memory. We also performed electrophysiological recordings *in vitro* in hippocampal slices prepared 6 and 9 months post-irradiation to evaluate excitatory synaptic transmission and plasticity. Next, we evaluated amyloid β (Aβ) deposition in the contralateral hippocampus and adjacent cortex using immunohistochemistry. In cortical homogenates, we analyzed the levels of the presynaptic marker synaptophysin by Western blotting and measured pro-inflammatory cytokine levels (TNFα, IL-1β, IL-6, CXCL10 and CCL2) by bead-based multiplex assay. TG mice performed significantly worse than WT mice in the WM. Irradiation of TG mice did not affect their behavioral performance, but reduced the amplitudes of population spikes and inhibited paired-pulse facilitation in CA1 neurons. These electrophysiological alterations in the TG mice were qualitatively different from those observed in WT mice, in which irradiation increased excitability and synaptic efficacy. Irradiation increased Aβ deposition in the cortex of TG mice without affecting cytokine levels and increased synaptophysin expression in WT mice (but not in the TG mice). Although irradiation with protons increased Aβ deposition, the complex functional and biochemical results indicate that irradiation effects are not synergistic to AD pathology.

## Introduction

The space radiation environment is characterized by the presence of charged particles dominated by protons and helium nuclei originating mainly from the solar activity and, in lesser amounts, by fully ionized nuclei of all heavier elements often denoted as high charge (Z), high energy (E) particle radiation (HZE) originating from galactic cosmic rays. Most radiation is effectively shielded by Earth’s magnetosphere, but during deep-space missions, such as the planned mission to Mars, astronauts’ exposure will be unavoidable. The charged particles deposit their energy along their tracks in the process defined by linear energy transfer (LET). When they traverse biological tissues, the intense ionization along the linear particle tracks may impart significant damage to cells and subcellular structures [1]. The mechanisms of damage to cells with complex morphology, such as neuronal cells with extended dendritic trees and axons [2], are not well understood. To characterize the effects of space radiation on the central nervous system (CNS), ground-based studies in animals have been conducted for more than two decades using charged particle accelerators. With our progressively improving understanding of the space radiation environment and radiation risks to the CNS [3], the focus of recent studies have mainly been put on relatively low doses of ≤1 Gray (Gy), reflecting the doses of charged particles expected during a mission to Mars [4–6]. Numerous experiments have confirmed that exposure to high-LET HZE particles is generally more detrimental than exposure to the more abundant, but less energetic protons [7]. However, recent studies have clearly shown that even <1 Gy proton irradiation [8, 9], or its combination with HZE particles [10] may produce significant deficits in learning, memory and executive functions [8, 11]. Although protons (together with helium nuclei) are the major constituents of the space radiation spectrum, their effects on the CNS, and in particular on electrophysiological properties of neurons, synaptic transmission and plasticity have not been fully tested.

Radiation damage to the CNS and age-related neurodegenerative disorders such as Alzheimer diseases (AD) share several pathological hallmarks, and both have been characterized as an accelerated aging-like process [12–14]. The common biochemical findings in AD that may lead to synaptic damage include increased oxidative stress [15], massive accumulation of pro-inflammatory cytokines [16–19], vascular damage [20], and impaired neurogenesis documented both in human AD [21] and in murine transgenic AD models [22]. Similar to the normal aging process and AD [23], increased oxidative stress has been reported in neuronal tissue after a single low dose exposure to ionizing radiation [24, 25], which has been associated with behavioral decrements [26]. Whereas the formation of reactive oxygen species peaks along the particle tracks within nano- to milliseconds after traversal through the CNS tissue, a lasting shift in the redox balance can be observed in neuronal cell lines *in vitro* [27], and in the hippocampus in *vivo*, indicating the presence of chronic oxidative stress [28]. Thus, in irradiated CNS, the surge of free radicals is likely the initial trigger leading to a wide spectrum of delayed neurological and cognitive decrements. Next, similar to AD, impaired neurogenesis in the hippocampal dentate gyrus is another common finding in irradiated rodent brain that may be associated with decrements in hippocampus-dependent memory and synaptic plasticity [26, 29–33]. Irradiation of the CNS also leads to the accumulation of pro-inflammatory cytokines within the CNS [34–38]. In AD, elevated brain levels of TNFα, IL-1 and IL-6 have been associated with severe cognitive decrements in animals and humans [16, 17, 39, 40]. Elevated levels of pro-inflammatory cytokines can impair hippocampus-mediated memory [39] and directly inhibit synaptic plasticity in hippocampal slices [41–44]. Furthermore, prominent vascular changes are found in both irradiated brains [45–47] and in AD [20]. Thus, the similarities between AD- and radiation-induced neuropathology suggest multiple common pathways that may be additive or synergistic [48].

The most significant structural marker of AD is the formation of senile plaques composed of insoluble amyloid beta (Aβ) peptide in several brain areas such as the cortex and/or the hippocampus [49, 50]. The Aβ deposition in AD is typically accompanied by variable degrees of cognitive, behavioral and electrophysiological decrements [23] that have been linked to synaptic damage in these brain areas. Although an increased Aβ deposition has not typically been observed in irradiated CNS, Cherry et al., found in APP/PSEN1 double TG mice predisposed to AD-related amyloidosis that a whole-body irradiation with ^56^Fe nuclei (1 GeV/n) caused elevation of brain Aβ levels, which they attributed to impaired Aβ clearance due to radiation-induced damage to the vasculature [51]. In addition to amyloidosis, others and we [52, 53] have confirmed that irradiation may accelerate other aspects of AD-related neuronal deficits, such as the synaptic dysfunction in the hippocampus. For example, we showed in TG APP23 mice that a single brain-only exposure to ^56^Fe nuclei (600 MeV/n) accelerated electrophysiological decrements in synaptic excitability, reduced GABA-ergic feedback inhibition and impaired the neuronal output in CA1 neurons of the hippocampus, which was likely associated with the progression of AD-like pathology [52].

Synaptic spines are particularly important cellular structures that are impacted in both AD and irradiated brain. They contain synaptic and extra-synaptic receptors and other molecular components regulating pre- to postsynaptic mechanisms of signal transmission, synaptic efficacy and plasticity. Thus, it has been pointed out that prior to neuronal degeneration, it is the impaired synaptic efficacy that may be responsible for primary memory deficits at the very early stage of human AD [54]. Reduced density of synaptic spines has been repeatedly described in human AD [55] and in TG animal models of AD [56]. Notably, reduced densities of synaptic spines were also recently described in irradiated animals [57], including animals irradiated with high-LET HZE particles [2] and protons [8]. In the mouse hippocampus, proton doses as low as 0.1–2 Gy reduced the density of immature dendritic spines and increased the levels of the phosphorylated form of GluR1 (a subunit of AMPA receptor that mediates fast synaptic transmission) in the dentate gyrus when measured 10–30 days after irradiation [8]. These molecular changes have been associated with behavioral deficits in tasks such as “novel object recognition” and “object in place” suggesting impaired hippocampal and prefrontal cortical functions [8]. Notably, mice overexpressing mitochondrial catalase (an enzyme that decomposes hydrogen peroxide) were protected from the effects of proton radiation, possibly due to attenuation of radiation-induced oxidative stress [58]. In addition to the glutamate synaptic receptors, expression changes of other synaptic regulatory proteins, such as those involved in presynaptic neurotransmitter release and/or in postsynaptic receptor scaffolding, are prominent features in AD [59] and have also been reported in irradiated brain. They are particularly important markers as their alterations may link a spectrum of electrophysiological, cognitive and behavioral deficits [23, 56, 60, 61] observed in both pathologies. Thus, reduced levels of presynaptic and postsynaptic proteins (e.g., synaptophysin and PSD-95, respectively) have been well described in patients with AD and in several murine transgenic models of AD [62]. Furthermore, in several animal models of AD, altered electrophysiological endpoints reflecting reduced synaptic transmission [56], excitability, or decrements in long-term potentiation (LTP) [63–65] have been reported. Earlier electron microscopy and morphometric studies in the rodent hippocampus exposed to accelerated argon nuclei demonstrated reduced synaptic density in the CA1 field of the hippocampus that correlated with behavioral deficits [66]. Exposure to iron nuclei (^56^Fe, 1.5 Gy, 1 GeV/n) reduced the levels of the presynaptic marker synaptophysin in the rat striatum, which also correlated with behavioral deficits [67]. Recently, reduction in presynaptic synaptophysin was confirmed in the hippocampus of mice exposed to low doses of protons along with increased postsynaptic marker PSD-95, but reduced dendritic complexity and synaptic spines numbers [8].

Due to these similarities and our previous findings, we set out to test the hypothesis that a single whole-body exposure to a low dose of protons may accelerate the onset of AD-like symptoms or aggravate the overall outcomes of the disease in a double TG murine model of AD. Similarly to Cherry et al., [51] we used a commercially available APP_swe_/PS1ΔE9 (APP/PSEN1) strain that contains a chimeric mouse/human form of amyloid precursor protein (APP) with the “Swedish” mutation (mouse APP695 harboring Aβ domain and mutation K595N and M596L), and lacks the exon 9 (ΔE9) in the presenilin 1 (PSEN) gene [68]. This model has been behaviorally and electrophysiologically well-characterized [69] and some functional changes have been observed to precede the biochemical findings [70], indicating that they may be the optimal experimental subject to detect and amplify any subtle, low-dose radiation-induced additive/synergistic effects. These TG mice were reported to produce increased Aβ isoforms that are detectable in the brain as early as 3 months of age, and Aβ deposition in the neocortex can be detected as early as 4–6 months of age [71]. Accordingly, APP/PSEN1 mice express increased levels of pro-inflammatory cytokines and chemokines, such as TNFα, IL-1β, IL-6, and CCL2 [72], an indication of neuro-inflammation. Although Cherry et al., found that irradiating these TG mice with ^56^Fe nuclei (1 GeV/n) increased Aβ [51], the authors did not test the effects of protons. Counterintuitively, the identical strain of TG mouse was recently used to demonstrate a beneficial effect of photon radiation on AD-type neurodegeneration and behavioral decrements. However, the authors used low LET radiation at relatively high doses (X-rays, ~10 Gy), well above those expected during space travel, and they only investigated short post-irradiation time periods [73]. None of the studies included wild-type (WT) counterparts in the experimental design, which limited the data interpretation. In our study, we included a limited cohort of WT mice to isolate the effects of irradiation and genotype on the functional (i.e., behavioral and electrophysiological) and biochemical endpoints. The inclusion of WT mice also facilitated the identification of the hypothesized additive/synergistic effects of irradiation in TG mice.

To identify the anticipated gradual, age-dependent functional decline, we used repeated behavioral tests in the same subjects, which helped to determine the progression of AD-related pathology [70]. We performed a series of behavioral tests in the water maze (WM) before irradiation and in the WM and Barnes maze (BM) at 3 and 6 months post-irradiation to evaluate spatial learning and memory. To further elucidate the synaptic mechanisms underlying the radiation-induced behavioral decrements, we performed a series of *in vitro* electrophysiological recordings in hippocampal slices 6 and 9 months after irradiation. In addition, at the post-irradiation time point exhibiting the most significant radiation-induced electrophysiological decrements, we performed immunohistochemical (IHC) analyses of amyloid β (Aβ) deposition in the hippocampus and adjacent cortical areas. In cortical homogenates only, we analyzed the expression of the presynaptic marker synaptophysin by Western blotting (WB) and measured levels of pro-inflammatory cytokines TNFα, IL-1β, IL-6, CXCL10 and CCL2 by bead-based multiplex assay (Luminex) to determine whether the irradiation aggravates synaptic and neuro-inflammatory hallmarks of AD.

## Materials and methods

### Ethics statement

This study was carried out in strict accordance with the recommendations in the Guide for the Care and Use of Laboratory Animals of the National Institutes of Health. Animal protocols were reviewed and approved by the Loma Linda University Institutional Animal Care and Use Committees (Protocol Number # 8100056). Terminal procedure was required for electrophysiological recordings followed by brain tissue collection. Mice were sacrificed at the age of 9 or 12 months under deep volatile anesthesia with 3.5% isoflurane and decapitated.

### Animals

Sixty-five male APP/PSEN1 TG mice (Jackson laboratories, strain no. 004462) and 16 male WT mice were included in the study. The WT mice were a strain of B6C3F1/J (Jackson, Stock#100010), in which females C57BL/6J (Stock#000664) were crossed with male C3H/HeJ (Stock#000659) mice. The animals were purchased at 2.5 months of age and delivered to the Loma Linda University (LLU) Animal Care Facility 10–14 days before irradiation. All mice were housed individually and kept at 21±1°C with 50±10% humidity and a light/dark cycle of 12 h. All experimenters for behavioral and electrophysiological parts of the study were blind for the dose group breakdowns until completion of the experiments.

### Experimental design and timeline

Mice were received from the vendor in 7 consecutive shipments (cohorts) separated by 3–4 weeks each about 10 days prior to irradiation. Upon arrival, TG animals in each cohort were randomly assigned to one of four groups, with doses of 0, 0.1, 0.5, and 1.0 Gy (final sample size N = 12-15/group). The cohort of 16 WT mice was divided into two groups exposed either to 0 or 0.5 Gy (N = 8/group). The WT mice were processed simultaneously with the TG mice; however, they exhibited some radiation-induced electrophysiological effects that we did not link with AD-like pathology (e.g., a suppressive effect of irradiation on epileptiform activity that was not observed in TG mice) and were reported, along with selected behavioral findings, elsewhere [9].

**Fig 1:**
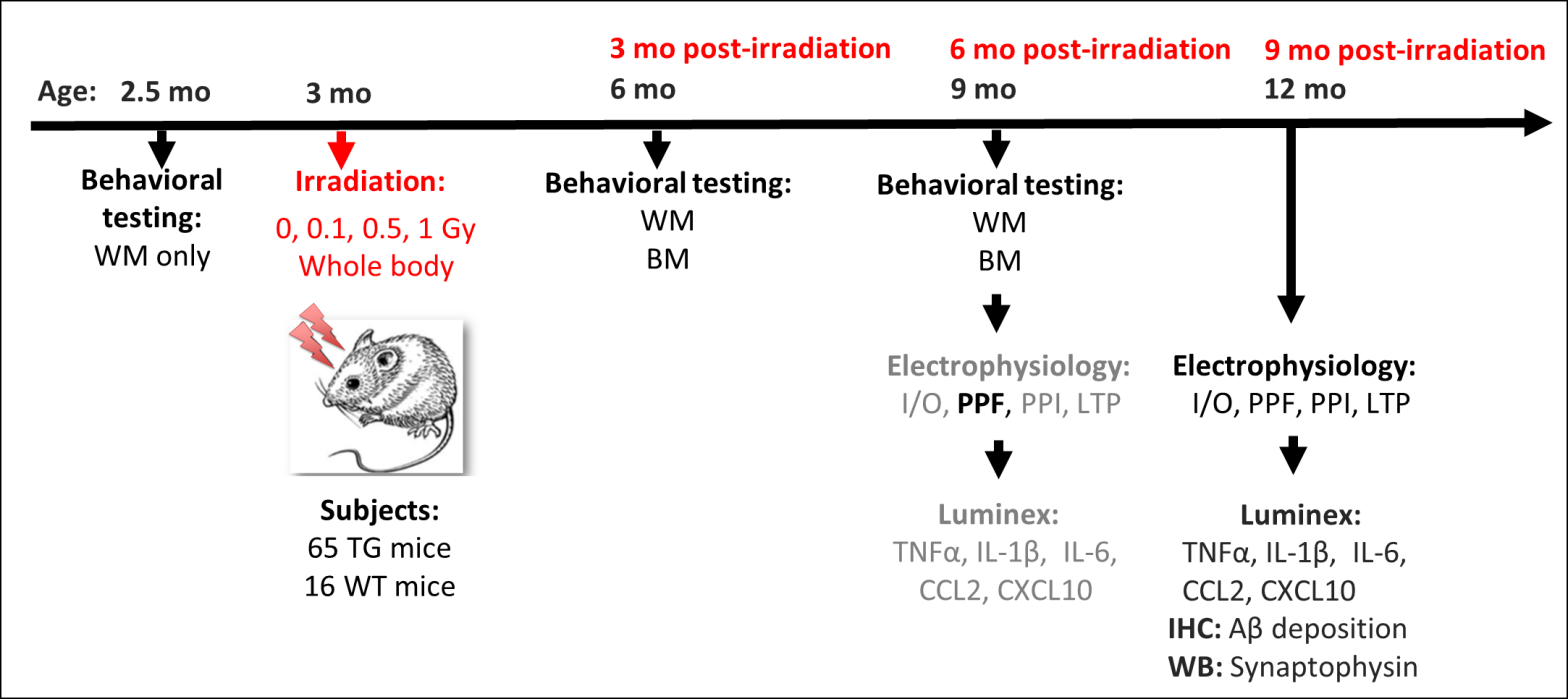
Timeline and design of the experiment. Sixty-five transgenic (TG) and 16 wild type (WT) male mice entered the study at the age of 2.5 months. Animals were behaviorally tested in the water maze (WM) before irradiation to determine the “baseline” performance. At the age of ~3 months they were irradiated with 0.1, 0.5, or 1.0 Gy of ~150 MeV protons. Post-irradiation, the behavioral testing consisted of the WM followed by the Barnes maze (BM). In half of the TG mice, the last behavioral testing was followed by *in vitro* electrophysiological recordings at 6 months post-irradiation. The second half of TG mice and all WT mice were electrophysiologically tested at 9 months post-irradiation. Electrophysiological protocols: (I-O) Input-Output tests; (PPF) Paired-Pulse Facilitation; (PPI) Paired-Pulse Inhibition; (LTP) Long-Term Potentiation. The grayed out methods at 6 months post-irradiation indicate the experiments that did not detect statistically significant radiation-induced changes and were not included in the manuscript. The exception was PPF (black), which showed significant age-related increase between 6 and 9 months post-irradiation. Cortices were used for Luminex cytokine assays. Immunohistochemistry (IHC) of Aβ deposition and Western blotting (WB) analyses of the presynaptic protein synaptophysin were performed at 9 months post-irradiation only.

Baseline spatial learning performance was assessed 7–10 days before irradiation using the WM only (Fig 1). Then, we repeated behavioral testing in the WM and, in addition, performed tests in BM at 3 and 6 months after irradiation (~6 and ~9 months of age). The pre-irradiation testing was necessary to determine the baseline behavioral responses before the onset of AD and for further comparisons of radiation, genotype and age effects associated with anticipated progression of AD at 3 and 6 months post-irradiation.

The first half of the TG mice were sacrificed within 14 days after the last training (6 months post-irradiation– 9 months of age), while the 2nd half of TG and all WT mice were kept to for another 3 months before sacrifice for *in vitro* electrophysiology (9 months post-irradiation—12 months of age). Note that we did not perform behavioral tests at this time point in order to keep an equal amount of behavioral training in both age groups and avoid potential differential effects on electrophysiology. Thus, the groups of TG mice used for behavioral tests were electrophysiologically investigated at either 6 or 9 months post-irradiation (N = 7–8 and N = 5–8 mice/radiation group, respectively). WT mice were tested only at the 9-month time point (N = 8 mice/radiation group). Electrophysiological tests were complemented with IHC assessment of amyloid deposition, expression of the presynaptic marker synaptophysin by WB and evaluation of brain cytokines by Luminex.

### Irradiations and dosimetry

TG mice were irradiated with doses of 0, 0.1, 0.5 or 1 Gy (whole body) at the age of ~3 months at The James M. Slater, MD Proton Treatment and Research Center at Loma Linda University. Animals were irradiated in the Bragg plateau region with a 20×20 cm beam of ~150 MeV/n protons (tuned energy) with 1 cm plastic modulation and < 3% variation in intensity across the central 18 cm [74]. Each mouse was placed in a ventilated acrylic box (3×3×8 cm) to minimize movement. Four mice at a time were aligned with the beam line with their heads located in the center of the trajectory and exposed to whole-body irradiation at a rate of ~0.45 Gy/min. The beam energy at target was reduced to ~142 MeV/n by traversal of the box with a resulting LET of ~0.57 keV/μm. The dosimetry was provided by the clinical control system calibrated to NIST-traceable ion chambers. The entire irradiation procedure lasted approximately 10 min per 4 mice. Non-irradiated (control) mice were placed in the clear acrylic boxes for the same amount of time as the irradiated mice to control for any potential effects of restraint-induced stress.

### Behavioral testing

#### Water maze

We performed behavioral testing in the water maze (WM) as described in detail previously [9, 75]. In brief, the WM was comprised of cued (day 1), spatial (days 2 and 3), and probe (days 2 and 3) tests. The maze consisted of a circular tub filled with water made opaque using white, non-toxic paint. A small platform onto which mice could step to escape the water was located in one of four quadrants. Each mouse was given 60 seconds to find the platform. Each swim path was recorded by an overhead camera connected to a computerized tracking system (Noldus Ethovision) to quantify total swim distance and other parameters. Ten trials were given in blocks of 5 each day (2 trials per block). Progressively less distance moved generally indicates better performance. The cued test is a control task that assesses sensorimotor (e.g., locomotion and vision) and motivational deficits that could alter performance on the spatial and probe tests. For this task, the escape platform was visible just above the water’s surface and there was a stick protruding straight out of the platform to make its location more salient. The platform location changed with each block, and mice were released nose against the wall directly across from the platform. The spatial task measures spatial learning ability. During this task, the escape platform was submerged just below the water’s opaque surface, and the mouse had to rely on spatial cues from around the room to find the platform, since it could not be directly seen. The platform remained in the same location throughout all 10 trials on the first day (Spatial 1), but changed to a different quadrant for all 10 trials on the second day (Spatial 2). The second day of spatial testing is considered the “reversal learning phase,” since it requires that mice disregard previously learned information (i.e., that the platform is in one location) and learn a new platform location. The probe test is a measure of spatial memory retention. We conducted the probe trial one hour after the completion of the last trial for each spatial test. For these trials, the platform was removed from the pool and we allowed mice to search freely for 60 seconds.

#### Barnes maze

The BM is a “dry land” spatial learning and memory task. The test included a circular table (110 cm diameter) with 20 holes along the outer surface and a hidden escape box located under one of the holes. Mice were placed in the center of the table and allowed to search for the hole to the escape box. The table was wiped with a 70% alcohol solution after each trial. We conducted the test over 3 days, with a procedure similar to the WM (i.e., Cued, Spatial, and Probe tests). Each animal received 5 trials per day for the cued and spatial tests. A stick was attached to the outside of the table to make the location of the escape box more salient during the cued test. If the mouse did not find the escape box within 5 min, the experimenter manually guided the mouse to the correct hole. For the probe tests, the escape box was removed and mice were allowed to search for 5 min. Distance moved and other parameters were assessed with an overhead camera, similar to the WM.

### Tissue collection

Mice were deeply anesthetized with 3.5% isoflurane and decapitated. The head was rapidly removed and immersed in ice-cold artificial cerebrospinal fluid (ACSF) containing 124 mM NaCl, 3.5 mM KCl, 1.25 mM KH_2_PO_4_, 2.0 mM MgSO_4_, 2.0 mM CaCl_2_, 26 mM NaHCO_3_, and 10 mM glucose with a pH of 7.4 and saturated with carbogen (95% O_2_ + 5% CO_2_). After chilling the head for 1.5 min, the brain was rapidly isolated and bisected along the mid-sagittal plane. The right hemisphere only was used for slice preparation and *in vitro* electrophysiology. The hippocampus was dissected free in ice-cold ACSF and chopped perpendicular to its longitudinal axis using a McIlwain tissue chopper. The adjacent cortex was flash frozen on dry ice for WB and the Luminex assay. The left hemisphere was fixed in 4% buffered paraformaldehyde solution and used for IHC analyses of amyloid plaque load.

### Electrophysiology

We used 4–5 adjacent slices (350 μm thick) from the rostral to rostro-medial part of the hippocampus for electrophysiological assessments. Slices were immediately transferred to a recording chamber, perfused with oxygenated ACSF at a flow rate of 1 ml/min, and incubated under submerged conditions at 33°C for at least 80 minutes prior to electrophysiological assessment.

We performed the majority of the recordings in the CA1 neuronal field, where synaptic responses were evoked by stimulation of the Schaeffer collateral/commissural pathway. In a limited group of slices we tested the long-term potentiation (LTP) in the CA3 neuronal field by stimulation of the mossy fibers as the main presynaptic afferent pathway. All other stimulation/recording parameters were identical for both investigated regions. We used 0.05 ms square constant-current pulses delivered through a concentric tungsten electrode. We recorded extracellular (field) synaptic potentials using glass microelectrodes (tip resistance 1–3 MΩ) filled with 3 M NaCl. Potentials were amplified (Axoclamp-2B, Molecular Devices, Sunnyvale, CA), digitized at a sampling rate of 10 kHz, filtered at 3 kHz and recorded for offline analyses using pClamp 10 (Molecular Devices, Sunnyvale, CA) and Mini Analysis (Synaptosoft, Inc.) software. All electrophysiological responses were further digitally filtered at 1 kHz low-pass filter to improve the signal-to-noise ratio.

We evaluated compound synaptic potentials in a manner similar to that described by Vlkolinský et al., with minor modifications [9, 52]. For most measurements, recording electrodes were positioned in the dendritic layer (*stratum radiatum*) to measure dendritic field excitatory postsynaptic potentials (fEPSP) and in the pyramidal layer (*stratum pyramidale*) to record somatic population spikes (PS). Dendritic potentials typically consisted of a small presynaptic fiber volleys (pV) followed by a large negative fEPSP, where the amplitude of the pV reflected the depolarization of the presynaptic terminals and the slope of the fEPSP reflected the magnitude of postsynaptic (dendritic) excitation. The fEPSP slopes were calculated from the initial negative linear segment of the traces using a least-squares regression analysis. Postsynaptic neuronal firing (“spiking”) of action potentials was evaluated by measuring the amplitude of the PSs. The PS amplitudes were calculated as the voltage difference between two positive peaks and the most negative peak of the trace.

To examine the full profile of excitability of CA1 neurons, we performed Input-Output (I-O) tests at incremental stimulation intensities (SI) ranging from 0.05 to 1.50 mA in 0.05 mA increments. These SIs were typically sufficient to evoke maximal postsynaptic responses determined by plateauing PS amplitudes. We measured increases in pVs and fEPSPs by the “dendritic” electrode, while the PS amplitude were derived from potentials recorded by the “somatic” electrode. Synaptic efficacy was calculated from the fEPSP slopes divided by corresponding pV amplitudes. Similarly, we calculated dendro-somatic functional coupling (denoted as EPSP-Spike [E-S] coupling) from PS amplitudes divided by corresponding fEPSP slopes. We also indirectly assessed the function of feedback inhibitory interneurons by applying paired-pulse stimulation at the SI evoking maximal PS amplitude and at inter-stimulus intervals (ISIs) of 6, 10, 20, 50, 100 and 200 ms. At 6, 10 and 20 ms ISI, this stimulation protocol typically led to the inhibition of the 2^nd^ PS that is denoted as paired-pulse inhibition (PPI) and was expressed as PS_2_/PS_1_ ratio. We also measured paired-pulse facilitation (PPF) and LTP of the fEPSP derived from dendritic synaptic responses recorded at reduced SIs. For PPF and LTP we reduced SIs to those evoking only 30–50% of the fEPSPs exhibiting contamination with PS (fEPSP at PS threshold). These responses were considered as “baseline response” for PPF and LTP tests. We monitored the baseline for stability using single pulses at 0.02 Hz for at least 10 min. PPF was then evoked by paired-pulse stimulation at 20, 50, 100, 200, and 400 ms leading to facilitated 2^nd^ fEPSP (fEPSP_2_) and expressed as the fEPSP_2_/fEPSP_1_. For LTP measurements, we recorded the baseline fEPSP (evoked by single pulses) for another 10–15 min. Then, we induced LTP by two trains of high frequency stimulation (HFS; 100 pulses at 100 Hz separated by 20 seconds at unchanged SI). Fifty seconds later, we resumed baseline stimulation and continued recordings for the next 60 min.

### Immunohistochemistry

Aβ deposition was analyzed by IHC methods at 9 months post-irradiation in TG mice only. The left hemisphere of the TG animals was fixed overnight with 0.1 M phosphate buffer solution (PBS) containing 4% paraformaldehyde (pH = 7.3), then washed 3×1 hour in PBS and submerged in 30% sucrose buffer solution at 4°C for 72 hours, followed by freezing on dry ice. The frontal regions of the brain were removed, then up to 90 coronal sections that contained the hippocampus were cut in the rostro-caudal direction into free-floating sections (20 *μ*m) at -20°C using a cryostat (Fisher; Leica CM1950; Leica Microsystems GmbH, Wetzlar, Germany). Three hemi-sections per animal, representing rostral, medial and ventral hippocampus were directly mounted on each glass slide (Gold Plus, Fisher) and 1–2 representative slides per animal were selected and stained with 6E10 antibody (mouse monoclonal antibody for Aβ N-terminus 1–16, 1:1000, catalogue #SIG-39320, Covance Inc., Princeton, NJ). Sections were blocked for 1.5 h in phosphate buffered saline with 1% bovine serum albumin and then incubated overnight at 4°C with 6E10 primary antibody. On the next day, sections were incubated for 2 h at room temperature with goat anti-mouse secondary antibody coupled with Alexa-Fluor 488 (1:500, Invitrogen, Carlsbad, CA). Slides were cover-slipped with anti-fading medium (Vectashield, Burlingame, CA) containing 4,6-diamino-2-phenylindole (DAPI).

Multiple images were taken of a single section to obtain complete pictures of the dorsal and ventro-temporal cortices and the hippocampus. Images were merged and subjected to threshold analysis using Mercator software (Explora-Nova, La Rochelle, France). The hippocampus and dorsal cortex (Bregma level—1.94 mm) were manually outlined into regions of interest (ROI). All values were collected using an epifluorescence microscope (BX41, Olympus, Center Valley, PA). The threshold and morphological user-defined parameters were manually selected to maximize visualization of staining in the ROIs and parameters were kept consistent for all animals. The program automatically counted positive staining and false positives were manually deleted. We evaluated the number of plaques in defined ROI, pixel density, and individual plaque areas (*μ*m^2^). We selected area as the most consistent measure of the plaque load and calculated the plaque area relative to the total area of a given ROI in each section (e.g., hippocampus), which was expressed as a percentage and compared across experimental groups.

### Western blotting and bead-based multiplex cytokine assay

We measured synaptophysin expression by WB and brain cytokines levels by bead-based multiplex assay (Luminex). We performed these measurements in the cortex since the hippocampal tissue was used for electrophysiological tests and IHC. Cortices were homogenized in tissue extraction reagent lyses buffer (Life Technologies, Grant Island, NY, #FNN0071) in an approximate ratio 1 ml to 0.1 g of the tissue. Homogenates were centrifuged at 13,000 rpm for 30 minutes at 4°C and supernatants removed for analyses of the total protein content. The supernatants were used for both WB and Luminex.

WB was performed by standard methods using the NuPAGE Bis-Tris 4–12% Mini Gel (Life Science Technologies) and 1.0 μg of total protein/10 μl per lane. Nitrocellulose 0.2 μm membrane was used for protein transfer (Bio Rad) using the XCell II SureLock blotting apparatus (Life Technologies). We used primary mouse anti-synaptophysin monoclonal antibody (Millipore/Chemicon #MAB368; dilution 1/20,000) and β-tubulin antibody (dilution 1:5,000) as a house keeping protein/loading control (Thermo-Fisher Scientific/Pierce # MAS-16308). We used goat anti-mouse IgG-horseradish peroxidase conjugated secondary antibodies for the detection (Santa Cruz Biotechnology #SC-2031, dilution 1/10,000). Chemiluminescence was measured by SuperSignal WestDura Extended Duration Substrate kit, 1 ml total per membrane (Pierce/Fisher #37071) after exposure to X-ray film (CL-XPosure Film Fisher #34091) for 1 minute.

Five pro-inflammatory cytokines were measured by the Milliplex map kit–mouse cytokine/chemokine magnetic bead panel MCYTOMAG-70K-05 (EMD Millipore, Billerica, MA) using a Luminex^TM^ 100 reader (Luminex Corporation, Austin, TX) according to the kit manufacturer’s guidelines. Brain homogenates were diluted to 100 μg of total protein/well; 50 μl total volume. The lower limit of detection of the assay was 2.2 pg/ml for interleukin-1β (IL-1β), 4.9 pg/ml for IL-6, 5.7 pg/ml for TNF-α, 1.5 pg/ml for CXCL10 (old nomenclature IP-10) and 6.59 pg/ml for CCL2 (old nomenclature MCP-1). Samples were assayed in duplicates.

### Statistical analysis

Behavioral tests: We used mixed ANOVA with two factors (genotype and radiation) and two repeated measures (trial and time-point) to assess performance of mice in the WM and BM. When genotype effects were found, we subsequently conducted mixed ANCOVA to control for the Cued test of the specific behavioral assay.

Electrophysiology: In I-O tests, we used linear mixed-models (LMMs), which provided several distinct advantages over repeated measures (RM) ANOVA for longitudinal data analyses. Unlike RM ANOVA, LMM does not drop data list-wise when some of the repeated measures are missing and there is no strict assumption of sphericity. The best model was selected using likelihood-ratio tests based on the lowest Akaike’s information criterion score [76]. The correlations between repeated measures residuals were accounted for with heterogeneous autoregressive, antedependence, or Toeplitz covariance structures, depending on the best-fit model. For PPF and PPI tests, mixed RM ANOVA was used, with inter-stimulus interval (ISI) set as the within-subject factor and radiation dose, genotype, and post-irradiation time point set as the between-subject factors. Greenhouse-Geisser correction was applied upon the violation of the assumption of sphericity assessed by Mauchly’s test.

IHC, WB and Luminex assays: Quantification of amyloid plaque load, changes in synaptophysin expression and expression of cytokines were evaluated by 2-Way ANOVA. Two-tailed Sidak’s or Dunnett’s was applied to all post hoc tests. Statistical significance was set at *p*<0.05.

Statistical analyses were conducted with SPSS (IBM SPSS Statistics for Windows, Version 22.0. Armonk, NY: IBM Corp) or SigmaPlot software (Systat Software, Inc., UK) and graphs were generated with Prism Software (GraphPad Prism for Windows, Version 7.02 GraphPad, La Jolla, CA).

## Results

All 65 TG mice were observed daily up to 6 or 9 months post-irradiation until they were sacrificed for *in vitro* electrophysiology. We observed occasional spontaneous deaths (for which we were unable to determine the cause) in the control group (2 out of 16 mice), 0.1 Gy (1 out of 16), but mainly in the 0.5 and 1 Gy dose groups (4 out of 16 mice in each dose group). However, the log-rank test did not confirm statistically significant radiation-induced mortality in any of the TG groups (S1 Fig). Due to this mortality, our sample size for later electrophysiological tests was reduced from N = 8 to N = 5 mice/group in 0.5 and 1 Gy groups at 9 months, with all other groups containing N = 7–8 mice/group. We did not observe any incidental deaths in WT animals.

### Behavioral effects of irradiation in TG and WT mice

**Fig 2:**
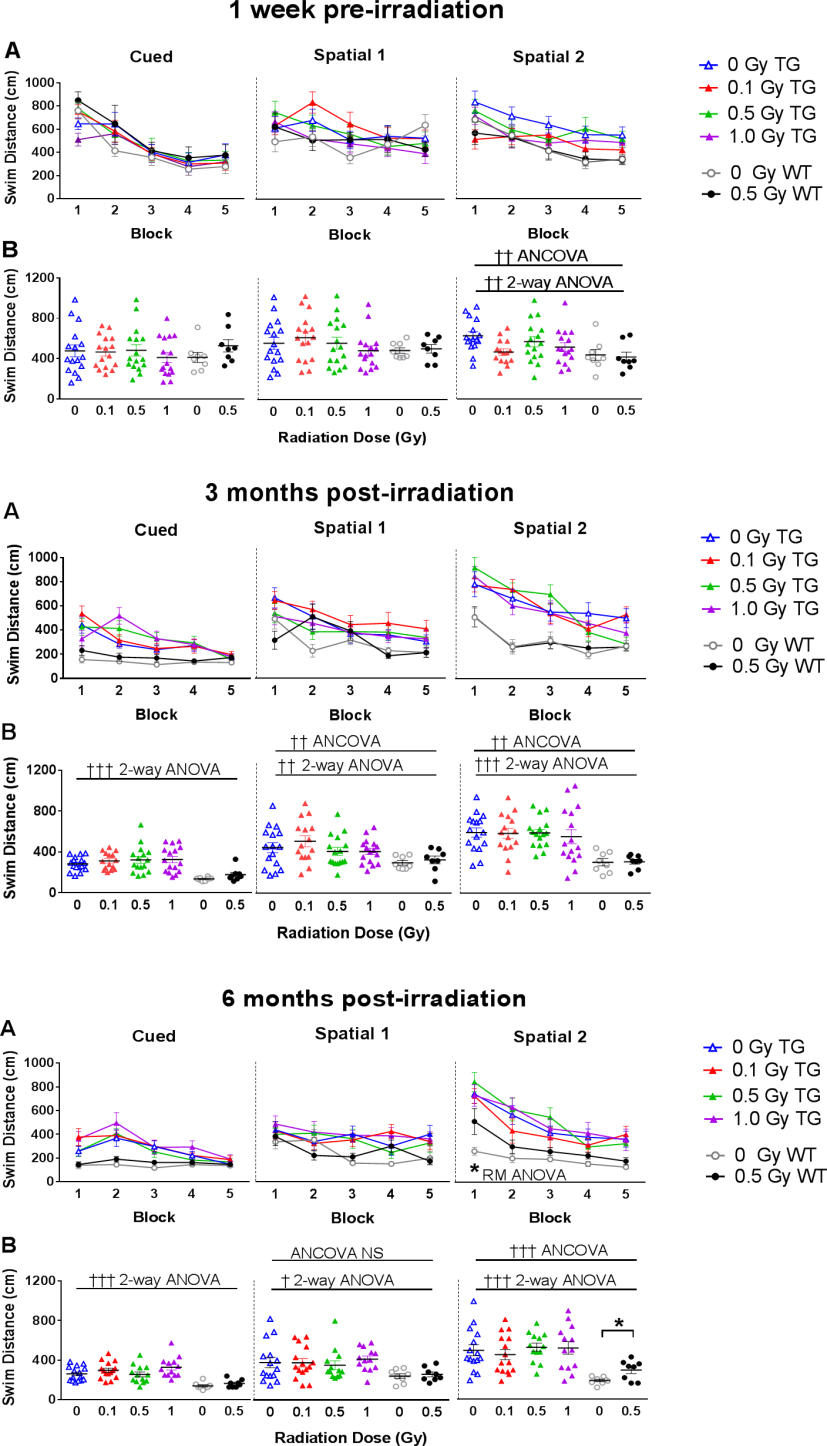
WM performance of TG and WT mice at baseline, 3 and 6 months post-irradiation. (A) Swim distance for each of the 5 blocks during Cued, Spatial 1 and Spatial 2 tests. (B) Swim distance averaged over the blocks. Upper panels A, B: Both the TG and WT mice exhibited gradually reduced swim distances in five successive blocks of each, Cued, Spatial 1, and Spatial 2 tests, suggesting that all groups were able to learn the task of locating the platform, which was most noticeable during baseline (pre-irradiation) testing. However, already at this time point (~2.5 months old) the WT mice performed significantly better than TG mice on the Spatial 2 (reversal learning) test. Middle and bottom panels A, B: At 3 and 6 months post-irradiation, we observed consistent genotype-related differences between TG and WT mice as the TG mice swam significantly longer in the Cued, Spatial 1 and Spatial 2 tests. When controlling for decrements in swim distance on the Cued test with ANCOVA at 6 months, the increased swim distance in the Spatial 2 test was still significant, which confirmed that the genotype-related decrement in TG mice are due to impaired hippocampus-dependent spatial memory. No significant radiation-induced differences were observed among the TG mice at any post-irradiation time point. However, irradiated WT mice searching for the relocated platform swam significantly longer distance during the Spatial 2 test at 6 months than the non-irradiated WT controls. This effect was most prominent during block 1 of that test, indicating radiation-impaired reversal learning in WT mice only (RM ANOVA; bottom panel A, right). TG mice: N = 12–15 mice/radiation group; WT mice: N = 8 mice/radiation group. Statistical analyses: Repeated measures ANOVA and ANCOVA, Open line—genotype effects ††† *p*<0.001, †† *p*<0.01, † *p*<0.05; Closed line—radiation effects * *p*<0.05. Data represent means ± SEM.

Results of the WM indicated that both the TG and WT mice exhibited improved performance (gradually reduced swim distances) in five successive blocks of each, Cued, Spatial 1, and Spatial 2 tests, suggesting that all groups were able to learn the task of locating the platform. However, we observed that WT mice performed significantly better than TG mice, which was most prominent in Spatial 2 tests. The genotype effects were detectable already at pre-irradiation baseline testing (Fig 2, upper panels A, B; 2-way ANOVA, Spatial 2, *F*_1,75_ = 9.29, *p* = 0.003) and became more prominent in all tests at 3 months post-irradiation (Fig 2, middle panels A, B; 2-way ANOVA, Cued, *F*_1,75_ = 26.85, *p*<0.001; Spatial 1, *F*_1,75_ = 7.90, *p* = 0.006; Spatial 2, *F*_1,75_ = 27.01, *p*<0.001). TG mice also performed worse than the WT mice in all tests at 6 months post-irradiation (Fig 2, bottom panels A, B; 2-way ANOVA, Cued *F*_1,68_ = 28.12, *p*<0.001; Spatial 1, *F*_1,68_ = 8.86, *p* = 0.004; Spatial 2, *F*_1,68_ = 23.05, *p*<0.001). Notably, WT mice outperformed TG mice in the Cued test at 3 and 6 months post irradiation. Therefore, we considered it important to confirm that differences in swim distance in spatial tests were not caused by sensory-motor or motivational decrements (typically revealed by the cued tests), which had been reported in this strain of TG mice, although at a much older age of 20–26 months of age [77]. Thus, we controlled for decrements in TG mice using the time-point’s respective Cued test data by ANCOVA and confirmed that baseline (pre-irradiation) Spatial 2 performance remained significant (ANCOVA, *F*_1,74_ = 9.70, *p* = 0.003). Similarly, at the 3 months post-irradiation time point ANCOVA confirmed that the deficits in Spatial 1 and 2 were still significant (Fig 2, *F*_1,74_ = 8.23, *p* = 0.005, *F*_1,74_ = 10.16, *p* = 0.002, respectively), which suggests genotype-related decrements in TG mice, most likely caused by impaired hippocampus-dependent spatial memory. At 6 months, ANCOVA confirmed the significance of such genotype-related decrements in the Spatial 2 (reversal learning) tests (Fig 2, bottom panels A, B; *F*_1,67_ = 15.52, *p*<0.001), but not in Spatial 1 tests (*F*_1,67_ = 2.70, *p* = 0.105). Although these behavioral deficits confirmed our experimental model of AD pathology in APP/PSEN1 TG mice (i.e., that WT mice outperform TG mice on most hippocampus-dependent spatial memory tasks), we did not identify any radiation-induced decrements in TG mice. Similarly, when we compared behavioral endpoints in WM across different time points, we did not identify any age-related decrements in either WT mice or in TG mice (data not shown). Importantly, in this extended statistical setting we confirmed the previously observed radiation-induced decrements in WT mice at 6 month post-irradiation with 0.5 Gy in the Spatial 2 test, which was most prominent during block 1 and indicated impaired reversal learning of the new platform location (Fig 2, bottom panel A; RM ANOVA, *F*_1,14_ = 7.76, radiation effect *p* = 0.015) [9]. No radiation effects were found in the Probe tasks reflecting unaltered spatial memory performance (Data not shown).

Last, all animals underwent testing in the BM at 3 and 6 months post-irradiation. At 3 months, significant genotype effects were observed on the Spatial 2 test only (RM ANOVA, *F*_1,71_ = 9.52, *p* = 0.003). At the 6 month time-point, we observed genotype effects on the Cued test (RM ANOVA, *F*_1,68_ = 12.33, *p*<0.001), but no significant Spatial 1 or Spatial 2 differences when controlling for these cued effects (ANCOVA, *p* = 0.072 and 0.112, respectively). Similar to the WM results, no radiation effects were observed at either time-point on the BM (S2 Fig). No differences were found on the Probe tasks (Data not shown).

### Electrophysiological effects of irradiation in TG and WT mice

In TG mice, we did not observe any radiation-induced differences at the 6-month time point. With the exception of increased PPF in TG controls at 9 months, we also did not observe any statistically significant age-dependent changes in TG mice between the 6- and 9-month time points. Therefore, we focused our statistical analyses of radiation effects detected in mice at the 9-month time-point.

**Fig 3:**
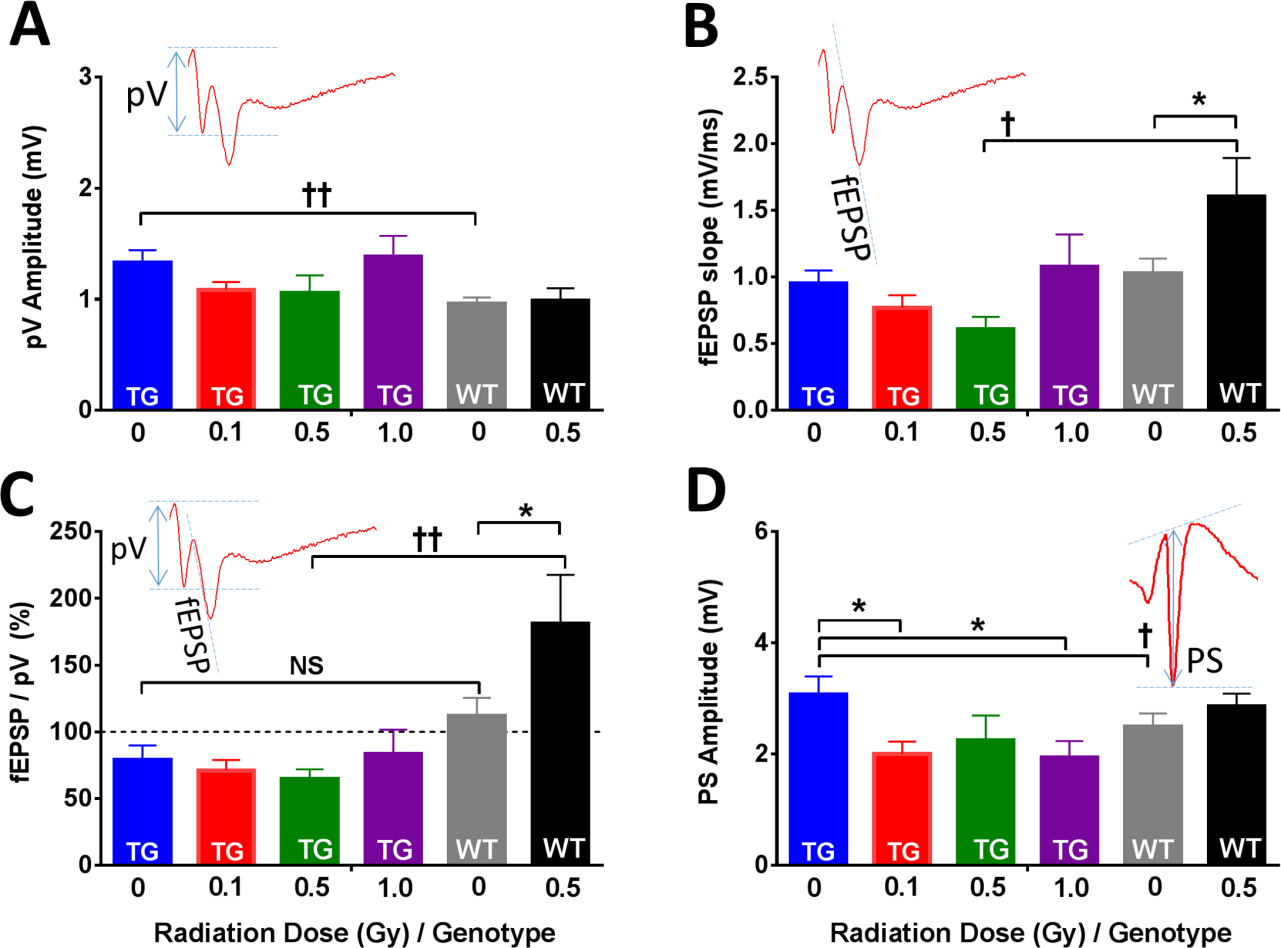
I-O functions in CA1 neurons in slices from TG and WT mice 9 months post-irradiation. (A) Radiation exposure did not significantly affect the presynaptic fiber volleys (pV) in either in TG or in WT mice. However, pV amplitudes were significantly elevated in TG controls when compared to WT controls. Inset: Original trace and measurement of pV amplitude is indicated with arrows. (B) Irradiation of WT mice (0.5 Gy) increased the slopes of the fEPSP by ~36% when compared to WT controls. In TG mice irradiated with 0.5 Gy, the fEPSP slopes tended to decrease indicating qualitatively different (and significant) radiation response from WT mice irradiated with 0.5 Gy. Inset: Original trace of the fEPSP recorded from the dendritic layer of CA1 neurons. Measurement of the initial linear slope of the fEPSP is indicated with a dashed line. (C) The synaptic efficacies in slices from non-irradiated WT control mice tended to be higher than TG controls, but the difference was not statistically significant (NS). The synaptic efficacy in WT mice was increased by irradiation at 0.5 Gy on average by ~39%. In TG mice, the synaptic efficacy was not affected by the exposure, but there was a statistically significant difference between slightly reduced efficacy in TG mice exposed to 0.5 Gy compared to WT mice irradiated with an equivalent dose. Inset: Original voltage trace indicating measurements of pV and fEPSP to compute synaptic efficacy for each slice. (D) Irradiation at 0.1 and 1 Gy significantly reduced the maximal PS amplitudes in TG mice by about ~35% indicating reduced output from CA1 neurons. When compared to WT controls, the PS amplitudes in TG control slices were significantly higher. Inset: Original voltage trace recorded from somatic layer of CA1 neurons and determination of the PS amplitude. TG mice: N = 12–13, 14–15, 7 and 10 slices *per* 0, 0.1, 0.5 and 1 Gy radiation group, respectively. WT mice: N = 16, 14 slices per 0 and 0.5 Gy radiation groups, respectively. Statistical analyses: Linear Mixed Models (LMM). Only post hoc tests are indicated: radiation effects * *p*<0.05; genotype effects † *p*<0.05, †† *p*<0.01. Data represent means ± SEM.

Input-Output (I-O) functions: In each slice, the recordings commenced with assessment of synaptic excitability in CA1 neurons, in which I-O functions were derived from presynaptic and postsynaptic components of compound synaptic potentials recorded at incrementally increasing stimulation intensities (SI). Note that the I-O functions were determined and statistically analyzed over the whole range of incremental SI from 0 to 1.5 mA (S3 Fig). For clarity, Fig 3 only shows the maximal magnitudes of the synaptic responses (Fig 3A–3D). Irradiation of TG mice tended to suppress pV amplitudes at 0.1 and 0.5 Gy (but not at 1 Gy), but the effect was not statistically significant. In WT mice irradiation had no effect on pV amplitudes indicating that presynaptic excitability was not impaired by the exposure. Further comparisons between TG and WT mice revealed a significant interaction between genotype and SI, as well as a significant genotype main effect (LMM, genotype×SI: *F*_24,116_ = 2.44, *p* = 0.001; genotype main effect: *F*_1,77_ = 6.05, *p* = 0.016). Thus, the pV amplitudes in slices of TG control mice were significantly larger compared to WT controls (Fig 3A; Sidak, *F*_1,77_ = 8.01, *p* = 0.006), but the difference was statistically significant only at SI>0.6 mA (S3A Fig). In TG mice we found no additional radiation-induced changes in pV amplitudes and we conclude that irradiation had no additive effect on presynaptic excitability.

In WT mice we previously observed that 0.5 Gy irradiation increased postsynaptic excitability (t-test, *p<*0.05, [9]). Here, we confirmed this radiation effect using LMM in an extended experimental setting that included both WT groups and four TG groups. Moreover, we detected a statistically significant interaction between irradiation and genotype, pointing towards qualitatively different radiation response on postsynaptic excitability in TG versus WT mice. While irradiation increased the postsynaptic excitability in WT mice, in TG mice excitability was not affected or tended to be reduced (LMM, dose×genotype: *F*_1,54_ = 4.09, *p* = 0.048). Significant differences in irradiated WT mice were observed at SI>0.9 mA, where irradiation increased fEPSP slopes by ~36% compared to control (S3B Fig; pairwise comparison *p* values ranged 0.002–0.038 at SI = 0.9–1.5 mA). Note that postsynaptic excitability never fully plateaued in irradiated WT mice, indicating that fEPSP in this group could be larger if higher SIs were applied (S3B Fig). When we compared TG and WT groups exposed to an equivalent dose of 0.5 Gy, the fEPSP slopes were significantly higher in the irradiated WT group (Fig 3B; Sidak, *p* = 0.036). Thus, irradiation appeared to impact postsynaptic excitability in a qualitatively different manner in TG compared to WT mice.

Proton radiation also differentially affected the synaptic efficacy in TG and WT mice. While irradiation significantly enhanced efficacy in WT mice it did not induce any changes in TG mice (main dose effect *F*_1,46_ = 0.85, *p* = 0.36). We identified a significant main effect of genotype (Fig 3C, *F*_1,46_ = 8.88, *p* = 0.005) and a significant interaction between genotype and irradiation (LMM, dose×genotype: *F*_1,46_ = 4.63, *p* = 0.037). Within the WT group, radiation increased synaptic efficacy on average by ~39% (Sidak, *p* = 0.013). Due to the bidirectional response to irradiation in TG and WT mice exposed to 0.5 Gy, there was a significant difference in synaptic efficacy between genotypes at 0.5 Gy (Sidak, *p* = 0.002).

The last step in the chain of signal transduction is the generation of action potentials by the postsynaptic CA1 neurons. Their summated synchronous spiking was measured as the population spike amplitude (PS; Fig 3D), which is considered the main neuronal output from the hippocampus. In the TG mice, irradiation at doses of 0.1 and 1 Gy induced significant decrements in PS amplitude, whereas in WT mice exposed to 0.5 Gy, the PS amplitude tended to increase (Fig 3D and S3D Fig). Thus, in TG mice, we identified a significant interaction between the radiation dose and SI (LMM, dose×SI: *F*_72,427_ = 1.51, *p* = 0.007) and radiation dose main effect (*F*_3,41_ = 2.98, *p* = 0.043). We further investigated the interaction with Sidak-corrected pairwise comparisons and found that the significant decrements in TG mice occur at SI>0.7 mA–approximately the point where I-O curves started to plateau (S3D Fig; *p*<0.05 for all post hoc tests in that range). At near maximal SI, the slices of both 0.1 and 1 Gy TG groups still exhibited significantly smaller PS amplitudes by ~35% compared to the control TG group. Although PS amplitudes in the 0.5 Gy group were also decreased, this difference was not statistically significant. Note that the decrease in postsynaptic firing was the only significant effect of irradiation on I-O tests in TG mice. When we compared TG controls mice with WT controls, the TG mice exhibited higher PS amplitudes (Fig 3D; Sidak, *p* = 0.029), and we identified interaction between genotypes and SI (LMM, dose×SI: *F*_24,237_ = 1.86, *p* = 0.011). The difference was detected specifically within the range SI = 0.4–1.0 mA (see S3D Fig; Sidak, *p*<0.05 for all post hoc tests in that range), rather than at the maximal SI, indicating differential recruitment of CA1 neurons by incrementally increasing SIs between both groups.

The E-S coupling was calculated as PS/fEPSP ratio, which reflects the ability of neurons to integrate the graded synaptic potentials from dendrites into action potentials by CA1 axons. While we previously showed that E-S was sensitive to irradiation with charged particles (e.g., 250 MeV/n Si nuclei, [78]) and protons [9], in this study the E-S was unchanged by either irradiation or genotype (data not shown), which was unexpected given that PS amplitudes were significantly reduced in irradiated TG animals (Fig 3D and S3D Fig).

**Fig 4:**
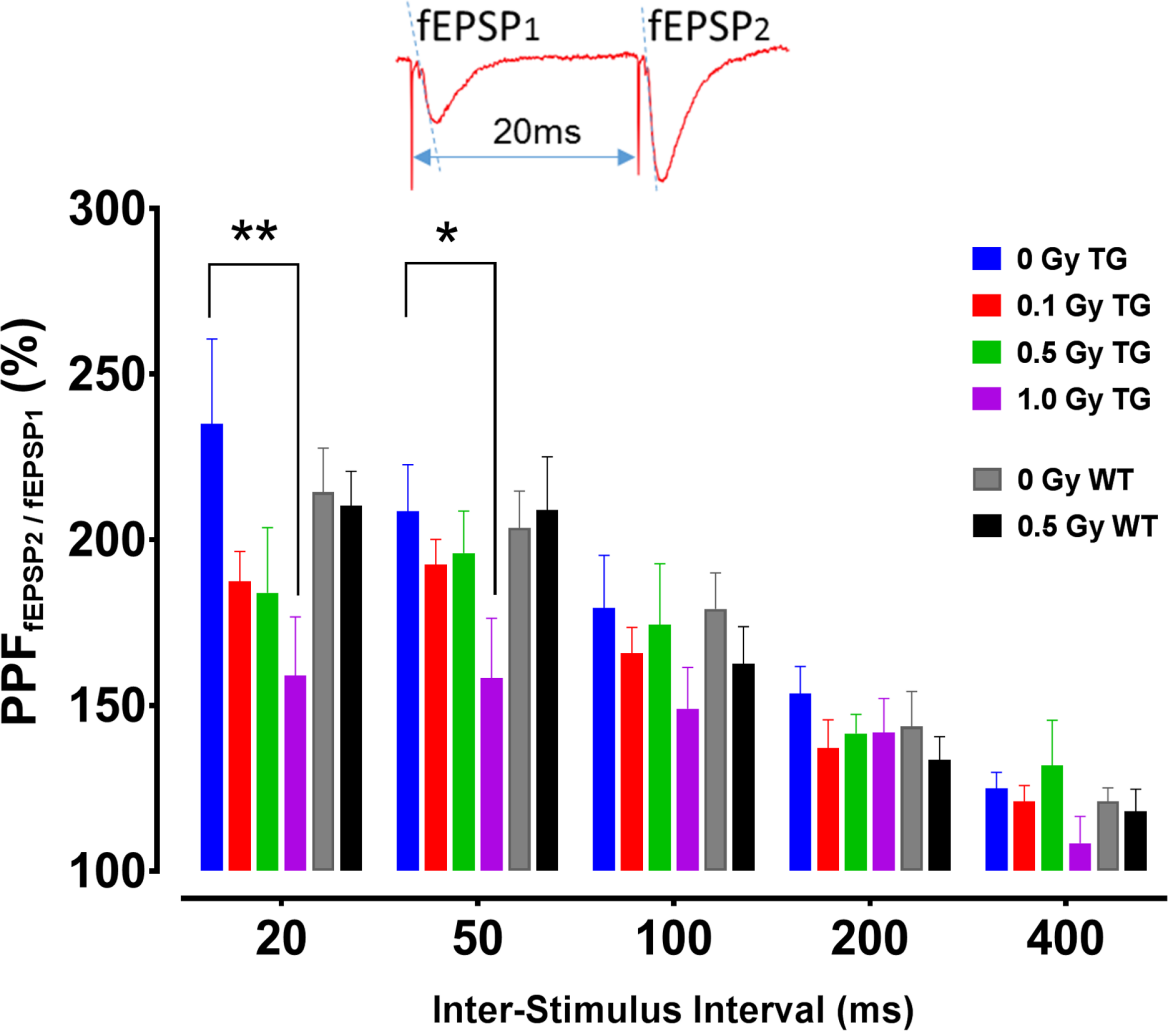
PPF in CA1 neurons of TG and WT mice 9 months post-irradiation. PPF ratios are shown at all tested inter-stimulus intervals (ISI) of 20–400 ms. Significant decrements in PPF were detected at 20 and 50 ms ISI in TG mice exposed to 1 Gy. Inset: Original voltage trace of PPF at 20 ms ISI showing facilitated slope of the 2^nd^ fEPSP (fEPSP_2_). Arrowheads indicate ISI between stimulation artifacts. Paired-pulse stimulation leads to facilitation of the fEPSP_2_. TG mice: N = 12, 15, 7 and 10 slices/radiation group; WT mice: N = 16, 14 slices/radiation group. Statistical analyses: RM ANOVA, Post hoc ** *p*<0.01, * *p*<0.05. Data represent means ± SEM.

**Fig 5:**
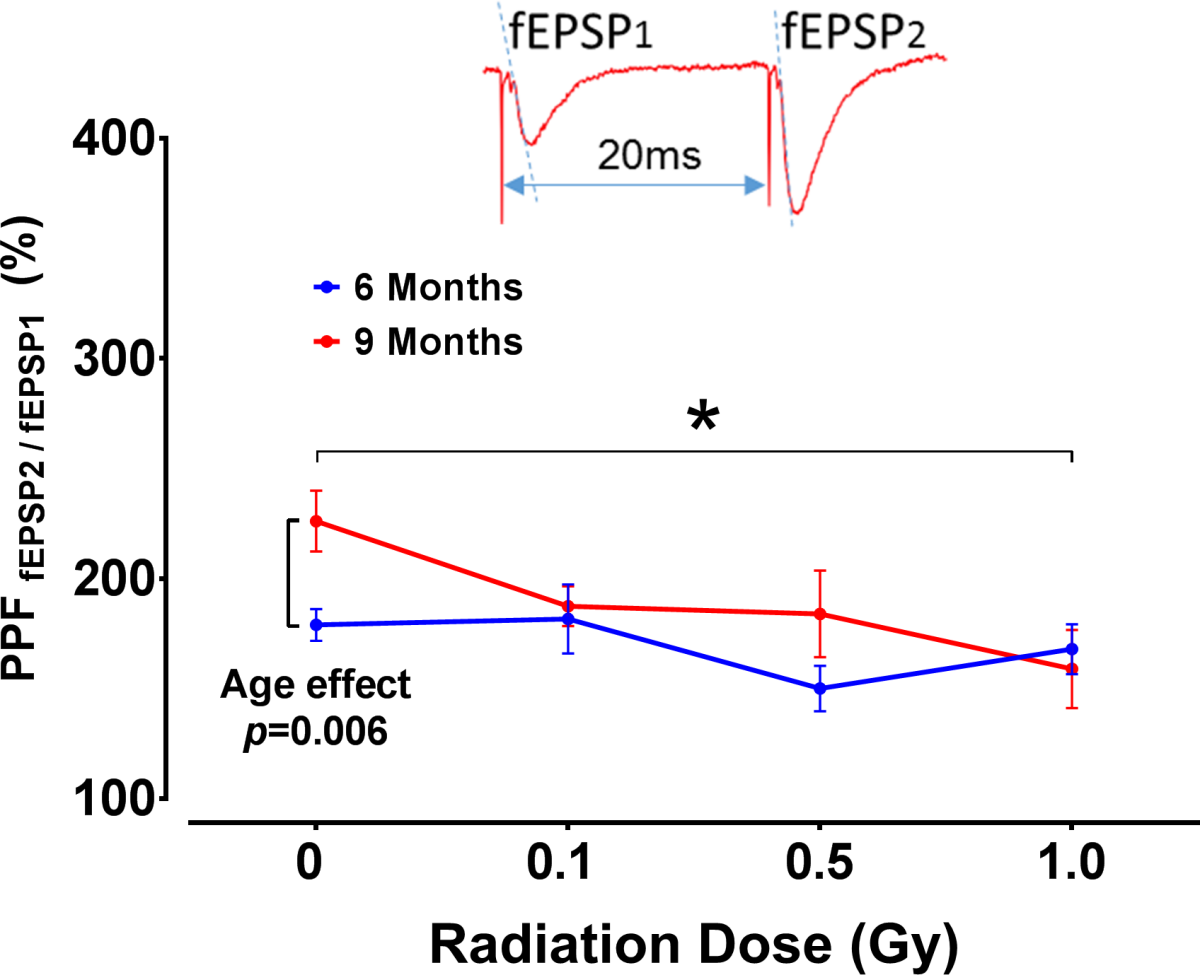
Radiation effects on age-related increase of PPF in TG mice. Comparison of PPF between at 6 and 9 months in TG mice revealed significant interaction between radiation dose and post-irradiation time point with enhanced PPF in 9-month TG control group only. Age increased PPF in control TG mice, and all doses of radiation reduced such PPF increases to the level observed in control TG mice at the 6-month time point. Significant reduction in PPF was detected at 1 Gy only. Inset: Original voltage trace at 20 ms inter-stimulus interval showing facilitated slope of the 2^nd^ fEPSP (fEPSP_2_). Arrowheads indicate stimulation artifacts. Note that the graph indicates PPF values acquired at 20 ms only (strongest PPF effect); however, the statistical model included PPF recorded at all ISIs. 6-month group: N = 11, 11, 8 and 10 slices/radiation group; 9-month group: N = 12, 15, 7 and 10 slices/radiation group. Statistical analyses: RM ANOVA, Post hoc tests * *p*<0.05, ** *p*<0.01. Data represent mean ± SEM.

Paired-Pulse Facilitation (PPF): PPF is a widely used, indirect measure of presynaptic neurotransmitter release probability (increased PPF indicates decreased release probability). In CA1 neurons, the PPF is typically most prominent at short ISI (20 ms) and becomes almost negligible at ISI≥400 ms (Fig 4). Irradiation (1 Gy) significantly reduced PPF at 20 and 50 ms ISI in TG mice at 9 months post-irradiation (Fig 4; RM ANOVA, radiation effect *F*_3,68_ = 3.19, *p* = 0.029; see also Fig 5) indicating increased probability of presynaptic glutamate release in these groups (TG 0 vs 1 Gy, Sidak, *p* = 0.019). There were no significant radiation effects in WT mice or differences between genotype (Fig 4).

The comparison of PPF between TG mice at 6 vs 9 months time points revealed an interaction between radiation dose and time point (age), with significant PPF changes in the control groups, but not in any of the irradiated groups (Fig 5; RM ANOVA, dose×age: F_3, 76_ = 3.39, *p* = 0.022; Post hoc at 0 Gy, *p* = 0.006), which confirmed an age-dependent decrease of presynaptic glutamate release previously reported in TG control mice. These data indicate that while age increased PPF in control TG mice at 9 months time point, all doses of radiation reduced (i.e., reversed) it to the level observed in control TG mice at the 6-month time point (Fig 5). Note that these PPF changes were the only age-related electrophysiological findings in TG mice.

**Fig 6:**
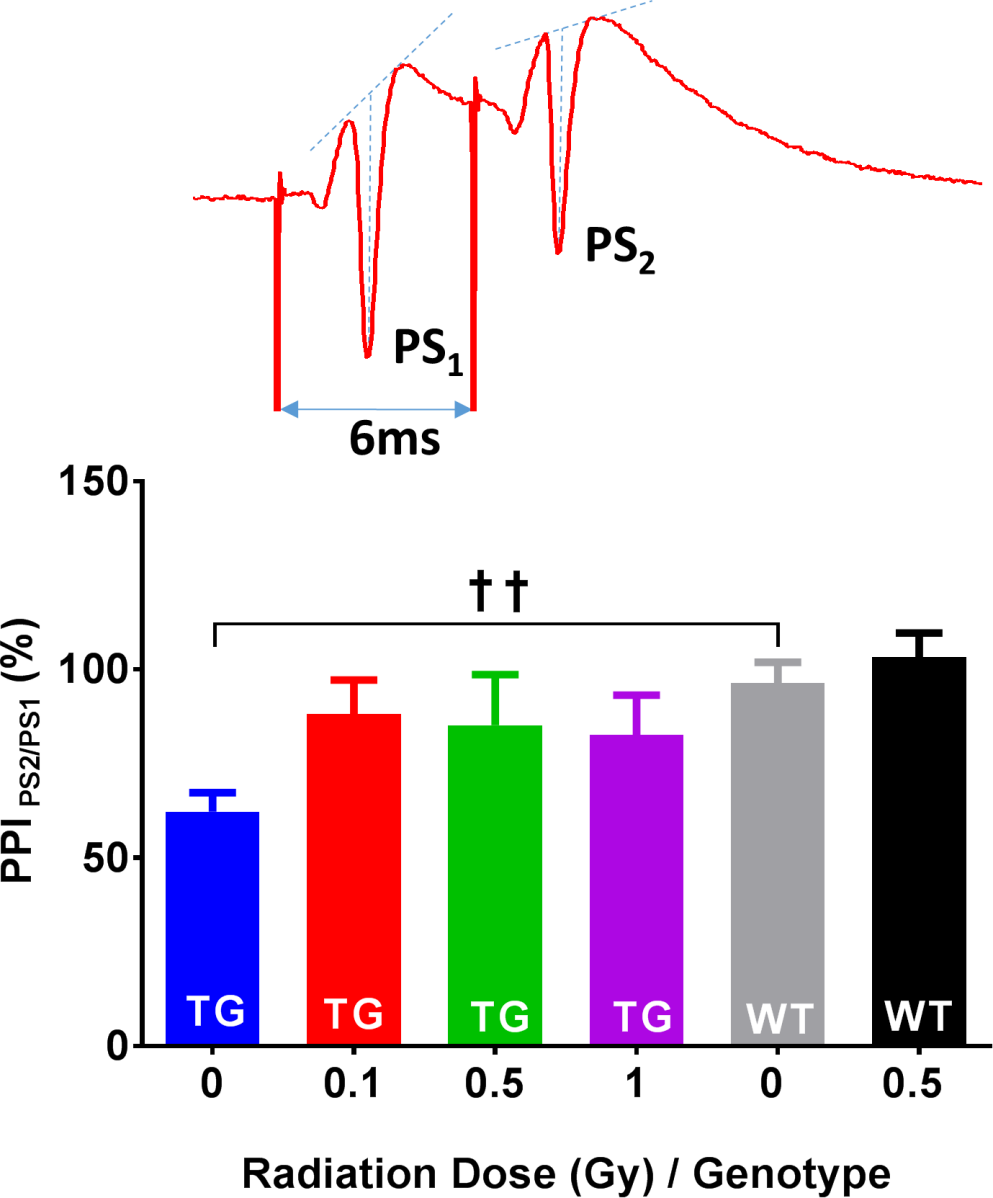
PPI in TG and WT mice 9 months post-irradiation. A comparison of PPI at 6 ms revealed stronger inhibition (~35% lower PS_2_/PS_1_ ratio) in TG control mice than in the WT control mice. Although all doses of radiation appeared to increase the PPI in TG mice, these increases were not statistically significant. Inset: Original voltage trace of PPI at 6 ms showing inhibition of the PS_2_ amplitude. Arrows indicate stimulation artifacts. Note that the bar graph indicates PPI values acquired at 6 ms; however, the statistical model included PPI recorded at all ISIs. TG mice: N = 12, 15, 7 and 10 slices/radiation group; WT mice: N = 16, 14 slices/radiation group. Statistical analyses: RM ANOVA, Post hoc †† *p*<0.01. Data represent means ± SEM.

Paired-pulse inhibition (PPI): PPI in CA1 neurons is a form of short-term neuronal plasticity mediated by a complex interplay between facilitatory synaptic/dendritic processes (PPF of the fEPSP) vs polysynaptic inhibitory GABA-ergic effects mediated mainly by feedback interneurons. The activation of feedback basket cells by the first action potential (PS_1_ at the first stimulation) can effectively reduce the number of firing CA1 neurons, which is observed as reduced second PS amplitude (PS_2_). Since the recruitment of feedback interneurons into PPI requires firing of action potentials in a large number of principal cells, the most prominent PPI is typically manifested at close to maximal PS_1_ amplitude and at short ISI (6–20 ms). Thus, the changes in PPI must be carefully interpreted, since they also depend on the relative magnitude of the initial response. The comparison of PPI between TG and WT controls at 9 months post-irradiation at all ISIs revealed a significant interaction between genotype and irradiation (*F*_2,132_ = 6.28, *p* = 0.003), and post hoc analyses revealed stronger PPI (about 35% lower PPI ratios, Fig 6; Sidak, *p* = 0.002) specifically in TG control vs WT control groups. Although all doses of radiation appeared to increase the PPI in TG mice, these changes were not significant (Radiation main effect: *F*_3,68_ = 1.36, *p* = 0.264).

In addition to excitability and short-term plasticity described above, we tested radiation effects on long-term synaptic plasticity in the CA1 (and in the CA3) neuronal fields, by measurement of LTP of the dendritic fEPSP, which we previously identified as an important radiation-sensitive endpoint known to be critical for cognitive processes. In spite of visible trends of enhanced LTP in CA1 neurons in all irradiated TG and WT groups, these measurements did not reveal statistically significant effects of irradiation, genotype, age, or post-irradiation time points (S4 Fig).

### Aβ deposition in TG mice

**Fig 7:**
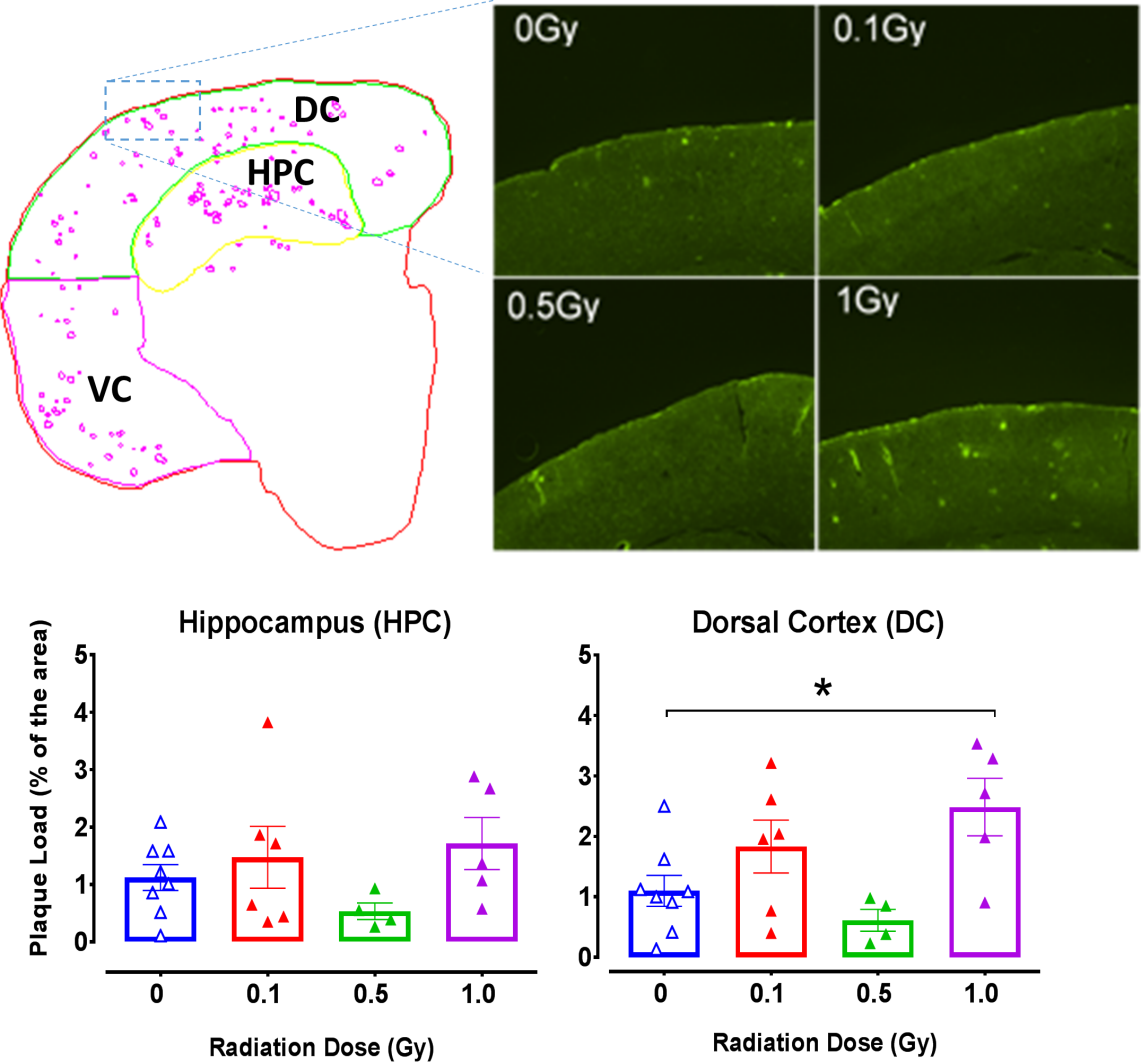
IHC analyses of Aβ deposits in TG mice 9 months post-irradiation. Top left panel: A representative illustration of a medial coronal section of the brain of a TG mouse divided into 3 areas of interest: hippocampus (HPC, dorsal cortex (DC) and ventral cortex (VC). The purple spots indicate the software’s built-in algorithm detection of amyloid plaques that was further visually confirmed by the experimenter. Top right panel: Original micrographs of cortical sections exposed to 0–1 Gy. Bottom panels: Relative numbers of plaque areas measured in the hippocampus and the dorsal cortex. Significant differences in Aβ deposition were detected between 0 vs 1 Gy groups in the DC and similar trends were observed in the HPC. TG mice only: N = 8, 7, 4 and 5 animals/radiation group. Statistical analyses: 1-way ANOVA, Post hoc * *p*<0.05. Data represent means ± SEM.

The 6E10 antibody recognizes the Aβ peptide at the epitope between amino acid sequence 1–16 and binds to both fibrillar and non-fibrillar isoforms. In all TG mice tested by IHC at 9 months post-irradiation, we observed prominent accumulation of Aβ. Furthermore, in the dorsal cortex of TG mice exposed to 1 Gy of proton radiation at 9 months post-irradiation we observed significantly more 6E10 antibody binding than in control mice (Fig 7; 1-way ANOVA, *F*_3,19_ = 4.39, *p* = 0.017, Sidak, *p* = 0.034). A similar trend was observed in the hippocampus.

### Cortical levels of presynaptic marker synaptophysin

**Fig 8:**
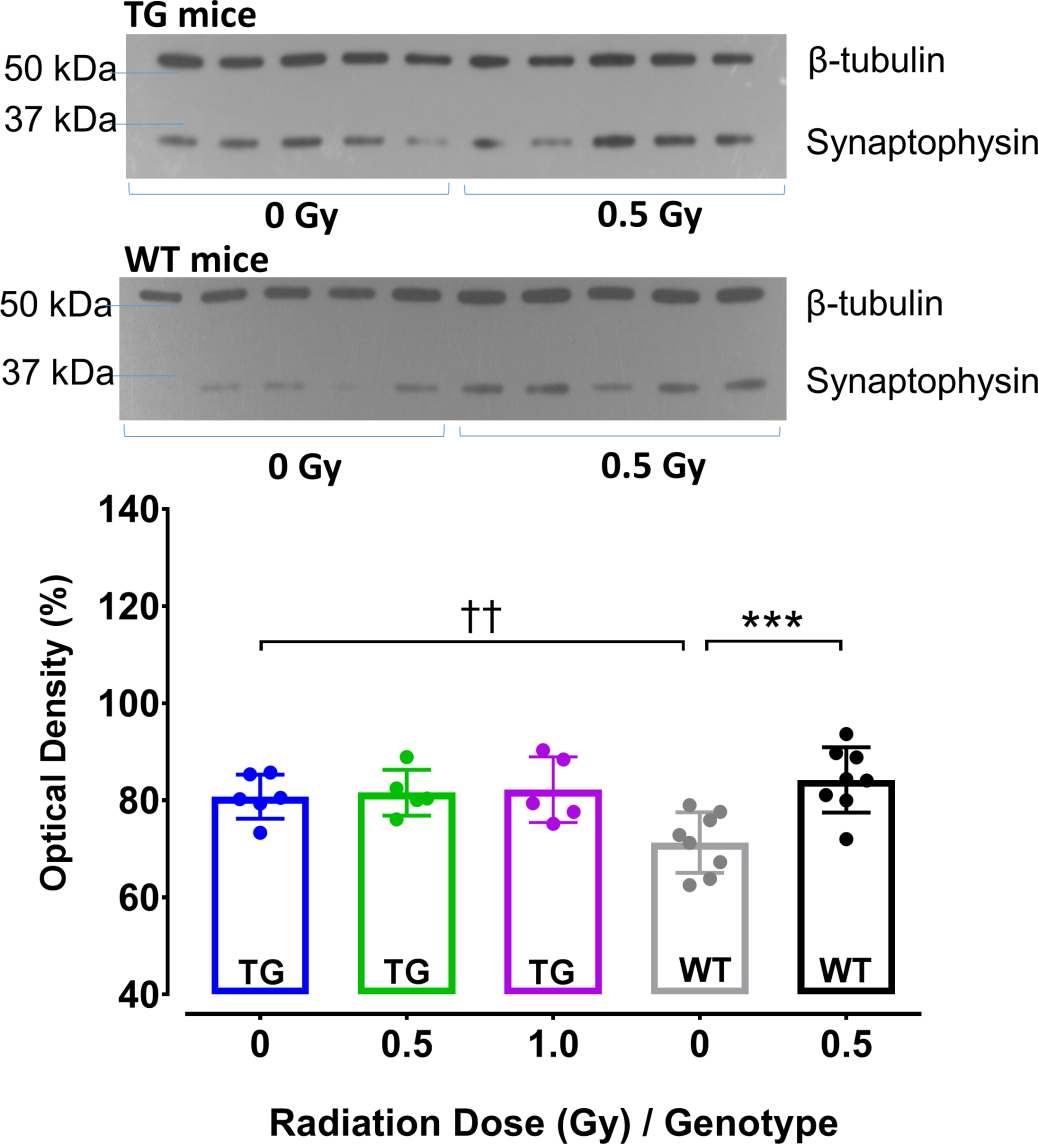
WB analyses of synaptophysin in the cortex of TG and WT mice 9 months post-irradiation. The average synaptophysin levels in TG control mice were significantly elevated when compared to WT controls. Irradiation of TG mice at 0.5 and 1 Gy had no effect on synaptophysin levels. In WT mice, irradiation significantly elevated synaptophysin to levels comparable to those observed in TG groups. Insets: Original images of WB gels prepared from TG (top panel) and WT (bottom panel) mice. Immuno-reactive bands optical densities indicate levels of synaptophysin (~38 kDa) and β-tubulin (~50 kDa) in cortical homogenates of control and irradiated 0.5 Gy mice. TG mice: N = 6, 5, 5 animals/radiation group; WT mice: N = 8, 8 animals/radiation group. Statistical analyses: 2-way ANOVA, Post hoc *** *p*<0.001 WT 0 Gy vs 0.5 Gy; †† *p*<0.01 WT vs TG controls. Data represent means ± SEM.

Alterations of synaptophysin levels have been reported in several different strains of APP TG mice [79], as well as in animals exposed to charged-particle radiation [67]. We tested whether such effects could be additive and related to changes in presynaptic glutamate release detected in electrophysiological experiments (i.e., PPF) described above. Thus, we analyzed expression levels of synaptophysin at 9 month post-irradiation and normalized to levels of β-tubulin (which was not affected by irradiation). In initial experiments with irradiated WT mice (tested at 0.5 Gy only), we noted a significantly elevated synaptophysin expression (*t*-test, t = 3.99 WT only, 0 vs 0.5 Gy; *p* = 0.014). The statistical analyses that included both genotypes revealed an interaction between genotype and radiation (Fig 8, 2-way ANOVA, genotype×dose, *F*_1,27_ = 6.65, *p* = 0.016) and significant radiation main effects (*F*_1,23_ = 4.39, *p* = 0.022). The post-hoc analyses confirmed radiation-induced difference in WT mice (*p*<0.001) and genotype-related difference between TG and WT controls (Fig 8, *p* = 0.007). The 0.1 Gy TG group was not included in WB analyses due to technical difficulties during sample preparation.

### Cortical levels of cytokines

**Fig 9:**
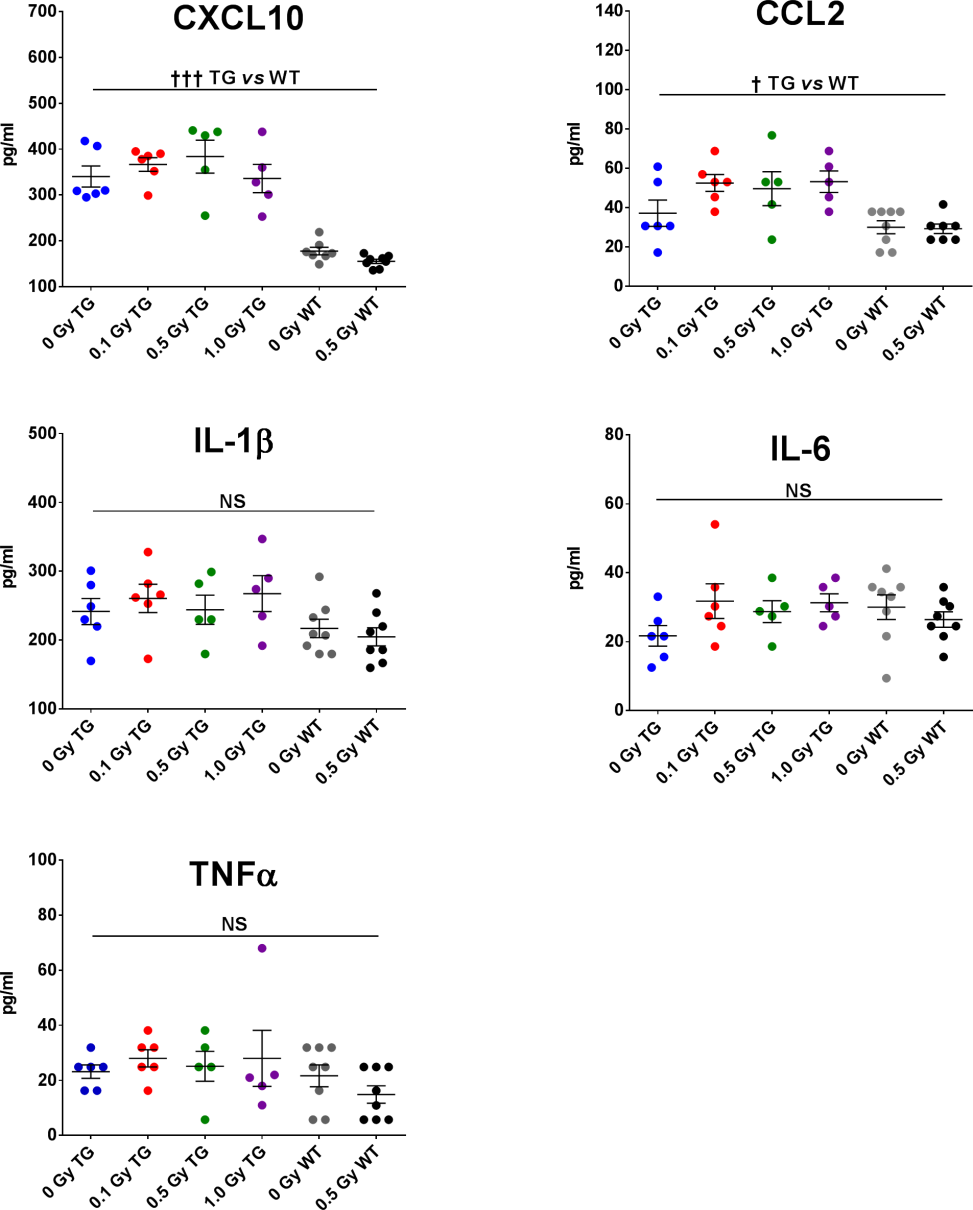
Cytokine levels in the cortex of TG and WT mice 9 months post-irradiation. Expression of CXCL10 and CCL2 was significantly higher in TG mice than in WT mice, but irradiation had no further effect on these cytokines. The differences in TNFα, IL-1β and IL-6 expression were not statistically significant (NS) in either TG or WT mice. TG mice: N = 6, 6, 5, 5 animals/radiation group; WT mice: N = 7, 8 animals/radiation group. Statistical analyses: 2-way ANOVA, genotype effect ††† *p*<0.001, † *p*<0.05. Data represent means ± SEM.

Neuroinflammation and elevated levels of pro-inflammatory cytokines are implicated in AD-related degenerative processes in APP/PSEN1 TG mice [72] and in irradiated brain [80, 81]. Thus, we expected that irradiation with protons would enhance their accumulation in the brains of TG mice. Because the elevated brain levels of TNFα, IL-1β, IL-6, CXCL10 (IP-10) and CCL2 (MCP-1) have been shown to modulate excitatory synaptic transmission and plasticity in the hippocampus [41, 42, 44, 82–84], we speculated that behavioral and electrophysiological alterations in TG mice were at least partly mediated by their increased levels. Thus, we compared the cortical levels of the above selected cytokines in TG and WT mice at 9 months post-irradiation. In general, cortical levels of all 5 markers tended to be higher in TG than in WT mice (Fig 9); however, this trend was statistically significant only for CXCL10 and CCL2, of which all TG groups showed elevated levels compared to WT (control and irradiated) mice (Fig 9; 2-way ANOVA; genotype effect CXCL10: *F*_1,32_ = 97.7; *p*<0.001 and CCL2: *F*_1,32_ = 6.93; *p* = 0.013). Irradiation in either TG or WT mice had no significant effect on cortical levels of these cytokines.

## Discussion

In this report, we have accumulated functional and biochemical evidence partially confirming our initial hypothesis that whole-body irradiation with protons alters hippocampal functions and may worsen some aspects of AD pathology in APP/PSEN1 double TG mice. To the best of our knowledge, this is the first report showing that protons, in addition to previously reported high-LET iron nuclei [51], can increase Aβ deposition in the brain of APP/PSEN1 TG mice. Although we have previously shown that 600 MeV/n iron radiation may accelerate the onset of AD-related functional decrements in TG APP23 male mice, in that study we only tested the electrophysiological endpoints and detected changes at doses >1 Gy [52]. Since the AD-related Aβ deposition was shown to be dramatically accelerated and further enhanced by the mutation of the APP cleaving enzyme presenilin 1 [68, 85–87], in this study we used APP/PSEN1 double TG mutants, as a presumably higher fidelity mouse model, to test for additive effects of less energetic (~150 MeV) proton radiation and at lower doses ≤1 Gy. We tested at time points/ages that were previously reported (depending on the specific endpoint) to be associated with progressive AD-related behavioral and electrophysiological alterations [70]. The irradiation was expected to aggravate behavioral performance, which was further expected to correlate with electrophysiological changes in the hippocampal synaptic transmission. However, the genotype-related behavioral decrements in TG mice were not affected by irradiation, thus we could not confirm the accelerated onset of AD by our behavioral analyses. Similarly, electrophysiological changes in TG mice were detectable only at 9 months after irradiation, which partly precluded analyses of accelerating effect on AD-related pathology. We avoided electrophysiological testing at earlier time points (e.g., 3 months post irradiation), because we did not anticipate exacerbation of AD-related synaptic decrements at the early age [70]. Our electrophysiological recordings *in vitro* revealed complex radiation-induced alterations in both TG and WT mice, but they did not indicate a unidirectional, synergistic effect with AD pathology (Table 1). Irradiation did not affect the expression of pro-inflammatory cytokines or the presynaptic protein synaptophysin in the cortex of TG mice, which also argues against additive effects of irradiation and AD. Thus, it is mainly the IHC finding of increased Aβ deposition at 1 Gy that supports our initial hypothesis on additive effect.

**Table 1:** Comparison of functional (behavioral and electrophysiological) effects of irradiation in TG and WT mice.

Endpoint	Test	Time point	Genotype effects	Radiation Effects
Behavior	Water Maze	Pre-irradiation	↑Swim distance in TG mice in SP2 (ANOVA & ANCOVA)	NA
		3 mo post-irradiation	↑Swim distance in TG mice in Cued (ANOVA), SP1 & SP2 (ANCOVA)	None
		6 mo post-irradiation	↑Swim distance in TG mice in Cued (ANOVA) & SP2 (ANCOVA). SP1 was not significant by ANCOVA	↑Swim distance in WT mice only in SP2 (Block 1)
	Barnes Maze	3 mo post-irradiation	↑Swim distance in TG mice in SP2 (ANOVA)	None
		6 mo post-irradiation	↑Swim distance in TG mice in Cued (ANOVA). SP1 & SP2 were not significant by ANCOVA	None
Electrophysiology	I-O curves–synaptic and CA1 neuronal excitability	9 mo post-irradiation	↑pV amplitudes in TG vs WT control mice ↓fEPSP slopes in TG vs WT mice at 0.5 Gy ↓Synaptic efficacy in TG vs WT mice at 0.5 Gy ↑PS amplitudes in TG vs WT control mice	↑fEPSP slopes in WT mice at 0.5 Gy ↑Synaptic efficacy in WT mice at 0.5 Gy ↓PS amplitudes in TG mice at 0.1 & 1 Gy
	*PPF–presynaptic glutamate release probability	6 & 9 mo post-irradiation	None	↓PPF ratio in TG mice at 1 Gy at 9 months. Note the suppressive effect on age-related increase in TG mice (6 vs 9 mo)
	**PPI–feedback inhibition	9 mo post-irradiation	↓ PPI ratio–stronger feedback inhibition in control TG vs WT mice	None
	LTP in CA1 and CA3 neurons	6 & 9 mo post-irradiation	None	None

↑significant increase, ↓significant decrease, NA—not applicable

*Note: ↓ PPF ratio indicates increased presynaptic glutamate release probability

**Note: ↓ PPI ratio indicates stronger feedback inhibition.

Note that significant genotype effects detected in WM and BM by ANOVA in Cued, SP1 and SP2 tests were further analyzed by ANCOVA to isolate the decrements (from Cued tests) in SP1 and SP2 tests only.

We performed this study with males only due to well-known fluctuations of behavioral and electrophysiological responses in female mice during their estrous cycle [88–90], which would increase the variability and reduce the statistical power of our experiment. Notably, the female mice in this TG strain were also shown to exhibit weaker radiation-induced amyloid deposition compared to TG males [51]. Although current NASA research solicitations encourage the use of both sexes in experiments, given the behavioral and electrophysiological instability in females, weaker radiation response and complex logistics of accelerator-based experiments, we did not include female mice in this study.

### Behavioral findings

#### Genotype-related behavioral changes in TG mice

The APP/PSEN1 TG mice were behaviorally characterized in multiple studies [91, 92] demonstrating amyloidosis as early as 4–6 months [68, 71], which was linked to behavioral decrements in spatial learning, including decrements in WM due to synaptic changes [69, 77, 93]. However, the functional changes in this strain of mice do not always temporally correlate with elevated Aβ levels in the brain and thus, a selection of appropriate behavioral (and electrophysiological) endpoints is critical for evaluation of progressive AD-related pathology. For example, Volianskis et al., demonstrated a complex relationship between behavioral decrements, Aβ deposition, and altered electrophysiological endpoints, where early decrements in reference memory and transient LTP (tLTP) preceded Aβ deposition, but they did not worsen with age. However, behavioral decrements in episodic memory that did progress with age and were accompanied with increased Aβ deposition, were not associated with electrophysiological decrements in excitatory synaptic transmission [70]. This complex relationship may explain our findings of early behavioral decrements in our TG mice as well as the fact that, in spite of increased amyloidosis at 1 Gy (together with the presence of electrophysiological alterations), we did not observe further behavioral decrements in TG mice exposed to radiation. Notably, Volianskis et al., identified an impaired tLTP between 3–12 months of age in this TG strain [70]. Since we only compared the TG and WT mice at the age of 12 months, it cannot be excluded that the early behavioral decrements in the WM were caused by similar early synaptic changes in hippocampal tLTP.

We observed WM decrements in TG mice in Cued tests, possibly indicating sensorimotor impairment or general associative learning issues related to the genotype. Indeed, Stover et al., reported on age-related visual disturbances in these TG mice due to retinal accumulation of amyloid; however, in much older mice than ours [77]. Volianskis et al., also pointed out impaired motor performance in the rotarod (but not in WM swim speed) [94]. These impairments may have contributed to decrements in our Cued tests. Nonetheless, when we controlled for decrements in Cued tests by ANCOVA, the overall data from our spatial tests at the 3 and 6 month time-points confirmed significant genotype-related impairments in spatial memory (Spatial 1 and 2 tests), which we attribute to AD-related hippocampal dysfunction. Interestingly, Savonenko at al., pointed out that their TG mice persevered in the search for an old platform location longer than the WT mice [69], which corresponds to the reversal learning deficit that we observed in our TG mice in Spatial 2 tests.

Spatial learning data from the BM indicated similar trends; however, they were less robust and ANCOVA did not find statistical significance. These results confirmed that use of the WM in our setting is a sensitive method to detect the AD-related functional decrements in mice, and would be expected to detect any additive radiation-induced damage.

#### Radiation-induced behavioral effects in TG and WT mice

Our behavioral tests demonstrated learning deficits in the TG mice relative to WT controls; however, they did not reveal any additive effects of irradiation in TG mice. Thus, the only radiation effect was detected in WT mice that exhibited impaired reversal learning at 6 months after irradiation with 0.5 Gy; this confirmed our previous conclusions derived from a more simple experimental design [9]. Namely, in a cohort of WT mice only, we previously observed that mice irradiated with 0.5 Gy of protons persevered in search for the removed platform and appeared to be slower in making a strategic switch, which resulted in significant increases in swim distance. Such behavior indicated impaired cognitive flexibility and reduced problem-solving ability due to irradiation. This radiation effect in WT mice is likely associated with electrophysiological changes in synaptic efficacy in hippocampal neurons (see Discussion below).

The absence of radiation effects on the behavioral endpoints in our TG mice contrasts with the findings of Cherry et al., who observed cognitive decline in an identical strain of TG mice at 6 months after irradiation with high-LET iron nuclei (0.10–1.0 Gy, 1 GeV/n). The authors described decrements in novel object recognition and contextual fear conditioning associated with increased levels of insoluble Aβ_1–42_ isoforms within the brain parenchyma [51]. Because both behavioral tests involve the hippocampus (in addition to other structures, such as the medial prefrontal cortex and the amygdala), the authors implicated hippocampal dysfunction. They also pointed out that endothelial activation and impaired clearance of Aβ oligomers, rather than neuroinflammation, might be the primary cause of increased Aβ deposition in their irradiated animals [51]. This is in agreement with our results of unchanged levels of brain cytokines (see below). Because the authors did not perform experiments in WT mice, they acknowledged that the behavioral effects might have originated from irradiation exposure only, rather than from the synergisms between the TG genotype and irradiation [51]. Our study with a limited cohort of WT mice did not reveal radiation-induced behavioral decrements, nor could we confirm synergistic effects of proton irradiation and AD-pathology. The discrepancy between the two studies suggests that irradiation with protons at similar doses is less effective than high-LET iron particles. Alternatively, their selection of behavioral tests may have been more sensitive in detecting the subtle radiation-induced decrements.

Finally, it should be noted that irradiation with relatively high doses of X-rays in identical, but older (64 weeks), male APP/PSEN1 TG mice was reported to have short-term beneficial effects on WM endpoints (and amyloidosis) in a recent study [73], but the potential molecular mechanisms of such beneficial effects were not elucidated. Similarly, one clinical case report described beneficial effects of repeated CT scans on the cognitive state of a patient with advanced AD, but potential protective mechanisms have not been identified [95].

#### Age-related behavioral changes in TG and WT mice

We expected that repeated behavioral tests after-irradiation would allow the identification of age- and AD-related decrements in TG mice, when neurodegenerative processes are not fully expressed and irradiation might impose additive effects. Volianskis at al., performed such repeated training using 6-arm radial WM and observed age–related decrements in WT and TG mice between the ages of 7 and 13 months; but genotype-related changes were detectable at the later age only [70]. Similarly, Savonenko et al., reported genotype-related decrements at 18, but not at 6 months of age [69]. Minkeviciene et al., observed first decrements in WM between 10–15 months of age [93]. When we compared performance in WM across different time points, we detected decrements in spatial learning in TG mice earlier than reported by the authors above; however, these decrements did not worsen between 6 and 9 months of age. Such absence of age-related decrements was unexpected. A possible explanation is that repeated training may lead to a training effect—a “recall” of the previously learned task—in addition to hippocampus-dependent spatial (re)learning at a given time point. Nonetheless, the hippocampal spatial relearning in the WM was still evident across sequential blocks of trials completed during the same day and could be inferred from negative slopes of the learning curves at each time point (Fig 2; Panels A).

### Electrophysiological findings

#### Genotype-related electrophysiological effects in TG mice

Enhanced brain levels of Aβ have been linked to reduced numbers of synaptic spines [96], impaired synaptic efficacy and compromised LTP [54, 97]. These deficits have also been experimentally triggered by brain injections of the soluble form of Aβ [96]. Comparison between our TG and WT control mice indicated distinct electrophysiological differences, including increased pV and PS amplitudes and stronger PPI in TG mice. To provide a mechanistic interpretation of these electrophysiological alterations, we suggest that increases in presynaptic excitability (pV recorded from Schaffer collaterals) and the enhanced neuronal spiking of CA1 neurons (PS amplitudes), which contrasted with stronger feedback inhibition (reduced PPI ratio, Table 1), may reflect a functional imbalance between excitatory glutamatergic CA1 neurons and inhibitory GABA-ergic interneurons within the local CA3-CA1 microcircuit. Such imbalance was reported in APP/PSEN1 TG mice and, if sufficiently strong, it could lead to impaired LTP [98]. Interestingly, such an imbalance in a CA1 microcircuit was recently reported in C57Bl6 mice irradiated with low doses of protons and was directly confirmed by paired-patch clamp recordings [99]. It is not clear if such microcircuit changes in our TG mice could be linked to behavioral decrements in WM.

Our TG mice did not exhibit significant decrements in LTP (recorded in both CA1 and CA3 hippocampal field). The absence of LTP changes in our TG mice is in accord with the results of Volianskis et al., using an identical strain of mice. They observed genotype-related decrements only in age-independent transient tLTP (between ages 3–12 months), but not the sustained form of LTP [70]. In WT mice, we measured LTP only at 12 months of age; hence, it is possible that we missed a critical time window to detect genotype-related decrements in tLTP reported for younger mice.

#### Radiation-induced electrophysiological effects in TG mice

Electrophysiological testing at 6 and 9 months post-irradiation revealed significant radiation-induced decrements only at 9 months only. In irradiated TG mice, the basal synaptic transmission appeared to be preserved, since presynaptic excitability (significantly enhanced in TG controls), synaptic efficacy and postsynaptic excitability were not affected by irradiation. The main impact of irradiation in the TG mice was observed on postsynaptic neuronal spiking in CA1 neurons and on altered presynaptic neurotransmitter release (i.e., reduced PS amplitudes and reduced PPF at 9 months post-irradiation, respectively). The genotype-related increase of neuronal spiking detected in TG control mice was inhibited by irradiation at 0.1 and 1 Gy, but not at 0.5 Gy. The reason for the absence of an effect at 0.5 Gy is not clear, but it might be attributed to the non-linear, stochastic nature of radiation responses commonly observed after exposures with charged particles [100]. The suppressive effect of irradiation on enhanced neuronal output in TG mice excludes synergistic effects of irradiation and AD. Nonetheless, the cellular/synaptic mechanisms of this radiation effect are intriguing and warrant further investigation since they appear to mitigate the small, but significant AD-related increase of CA1 neuronal output. The findings of reduced neuronal output from the hippocampus are similar to those that we previously observed in APP23 TG mice or immunologically-challenged C57Bl6 mice, both exposed to 600 MeV/n iron ions [52, 101]. We also observed reduced neuronal output in WT C57Bl6 mice 3 months after irradiation with 1 Gy of 600 MeV/n silicon ions [78] indicating that charged particle radiation may selectively impact the function of the axon hillock of CA1 neurons, where action potentials are preferentially generated.

Irradiation of TG mice at 9 months (but not WT mice) significantly reduced PPF (at 1 Gy only) indicating increased glutamate release from presynaptic terminals [102–104]. To the best of our knowledge, this is the first electrophysiological finding of radiation-altered PPF; however, enhanced spontaneous presynaptic glutamate release can be also inferred from our whole-cell patch-clamp studies in the proton-irradiated CA1 neurons [105] and iron-irradiated granular cells of the dentate gyrus [74] that exhibited increased frequencies of miniature AMPA-receptor mediated excitatory postsynaptic currents at 1 Gy. Interestingly, irradiation with iron ions at doses <1 Gy was previously reported inhibiting the evoked, readily releasable vesicular (glutamate) pool from rat hippocampal synaptosomes [106].

Since PPF in our WT mice was not affected by radiation exposure (tested at 0.5 Gy only), it appears that the increased Aβ levels in TG mice, which are known to interfere with presynaptic glutamate release and PPF [107], play the key role for the full expression of this radiation effect.

#### Age-related electrophysiological changes in TG mice–the effect of irradiation

When we compared PPF in TG mice at 6 versus 9 months post-irradiation time points, we identified significant age-related increases, which confirmed the previously reported observation on reduced presynaptic glutamate release reported between 7–17 months of age in identical TG mice [93]. It should be noted however that increased PPF in our TG control mice at the 9 month time point likely reflects an age–related alteration, rather than genotype related change (i.e., not a function of elevated Aβ levels), since it was not different from the PPF in WT control mice at the same age. Nonetheless, the elevated levels of Aβ in TG mice may somehow mediate the expression of the radiation-induced effects on neurotransmitter release. Thus, we found that age-related inhibition of neurotransmitter release in TG control mice was restored by irradiation to the level observed in younger TG animals (Fig 5). This radiation effect on presynaptic glutamate release in AD-prone subjects is intriguing and warrants further investigation.

#### Qualitative differences in electrophysiological responses to irradiation in TG versus WT mice–implications for behavior

**C**omparisons of electrophysiological changes in TG versus WT mice at 9 months post irradiation revealed several qualitative differences in response irradiation (see Table 1). Mainly:

1. WT mice in the 0.5 Gy group exhibited increased postsynaptic excitability and synaptic efficacy, which corroborated our previous results in CA1 neurons exposed to high-LET iron ions (4 Gy) and in the mouse dentate gyrus granular cells exposed to protons (1 Gy) [74]. However, in irradiated TG mice such effects were either absent or, in the group exposed to 0.5 Gy, a decrease of both synaptic parameters was detected (Fig 3 and S3 Fig);
2. The irradiated TG mice only exhibited significant decrements in neuronal output, suggestive of qualitatively different cellular site of radiation-induced effect in TG mice (e.g., the axon hillock of CA1 neurons) versus WT mice (dendritic/synaptic region);
3. Irradiation affected presynaptic glutamate release in TG, but not in WT mice.

To link the electrophysiological changes with behavioral decrements in WT mice, we suggest that the radiation-induced increase of synaptic excitability and efficacy may have interfered with long-term depression (LTD) in the hippocampus, which was shown to be specifically required for reversal learning in the WM [108, 109]. Alternatively, it may have saturated other forms of synaptic plasticity in the hippocampus (and other brain areas), and thus impaired spatial learning [110], even though such saturation was not detected in our LTP tests.

In TG mice we did not detect radiation-induced electrophysiological decrements at 6 months post-irradiation, which is consistent with the absence of radiation effects in behavioral tests. However, 3 months later, we uncovered radiation-induced decrements in neuronal output and glutamate release, indicating either that: (i) the irradiated neuronal network undergoes slow progressive change that manifests at or beyond 9 months; (ii) subtle synaptic changes were insufficient to affect behavioral response, or (iii) a specific time interval between last behavioral testing and *in vitro* electrophysiology was required to unmask radiation-induced electrophysiological decrements. All these possibilities may be responsible for lack of correlation between behavioral and electrophysiological results in TG mice. How would the electrophysiological decrements in neuronal output and glutamate release in irradiated TG mice translate to behavioral changes beyond >9 month-time point is unknown, but warrants further investigation.

In all, the electrophysiological recordings of synaptic excitability indicate qualitatively different responses to irradiation in TG versus WT mice and radiation effects do not seem to be additive or synergistic with AD-related pathology. We further conclude that at ≤1 Gy, the radiation-induced electrophysiological changes in our TG mice are relatively subtle. It is possible that they are compensated by the plastic nature of the neuronal network and thus, they may not necessarily translate into significant behavioral decrements.

### Biochemical findings

#### Aβ deposition in TG mice

APP/PSEN1 TG mice exhibit early amyloidosis as early as 4–6 months of age, reported to be associated with neuroinflammation and accumulation of cytokines [68, 71, 72]. Cherry et al., reported that high-LET iron radiation at doses of 10–100 cGy increased brain levels of soluble Aβ_1–42_ by 10–15% in male APP/PSEN1 TG mice (but not in females) at the age ~9 months, but amyloidosis was ascribed to endothelial dysfunction (reduced clearance of Aβ) rather than to enhanced neuroinflammation [51]. Our IHC experiments confirmed that proton radiation enhances Aβ deposition in the cortical areas with the same trend in the hippocampus. Similar to Cherry et al., we suggest that the radiation-enhanced Aβ deposition was not due to neuroinflammation, since we did not observe radiation-induced changes of pro-inflammatory cytokines in TG mice. Instead, increased amyloidosis is perhaps linked with vascular decrements [111] and leading to impaired transport of Aβ from the brain [51], which warrants further testing in these mice.

The APP/PSEN1 TG mouse is primarily a model for amyloid-induced of AD [112], Tau pathology is also important hallmark of AD, but appears to be less prominent in this TG model and delayed relative to Aβ deposition [113]. Nonetheless, tau phosphorylation and formation of neurofibrillary tangles may be affected by irradiation [114]. We did not evaluate tau pathology, but such measurements may reveal radiation response in appropriate TG strains [115], and warrant future testing.

#### Cortical synaptophysin levels

Since we observed radiation-induced decrements in PPF in TG mice at 9 months post-irradiation, we tested whether the functional changes could be associated with altered expression of the presynaptic protein synaptophysin. The hippocampal tissue in our experiments was used for electrophysiology and IHC, therefore we measured synaptophysin (and cytokine levels, see below) in homogenates of adjacent cortical areas. In murine TG models of AD the levels of synaptophysin in the hippocampus are typically reduced [79, 116], while in human AD they were reported to be either reduced [62] or unchanged [117]. Unexpectedly, the synaptophysin levels in our TG mice were higher than in WT controls, and they were not affected by the irradiation. Moreover, in WT mice exposed to irradiation, the expression of synaptophysin was significantly increased, rather than decreased. Such increase would be expected to be associated with altered PPF, which was not the case in irradiated WT mice. The discrepancies between radiation effects on the synaptophysin levels and PPF in our TG and WT mice are puzzling, but McMahon et al., have shown that synaptophysin might not be essential for PPF (or LTP) [118], thus future studies will be directed to other presynaptic markers.

### Cortical cytokine levels

The APP/PSEN1 TG mice used for this project express increased levels of TNFα, IL-1β, IL-6 from 8 to 10 months of age, associated with activated microglial cells and astrocytes [72]. In addition, increased brain levels of the pro-inflammatory chemokine CCL2 (MCP-1) are known to accelerate microgliosis and Aβ deposition (together with TNFα) in this mouse model [119]. We also investigated brain levels of the pro-inflammatory chemokine CXCL10, which we found previously to impact hippocampal PPF [44]. The common feature of these five biomolecules is their known inhibitory effect on hippocampal plasticity or basal synaptic transmission [41, 42, 44, 120]. We hypothesized that irradiation would enhance the brain cytokine levels in the TG mice and impact synaptic functions. However, at either 6 (data not shown) or 9 months post-irradiation, we did not detect any significant radiation-induced increases indicating that radiation did not enhance neuroinflammatory response in TG mice. Notably, fluctuations in radiation-induced cytokine response have been well documented [121, 122], which in future studies may require their measurements at multiple post-irradiation time points.

### Conclusions

We confirmed our initial hypothesis that whole-body irradiation with protons, similar to high-LET particles, would increase Aβ deposition in the brain and thus aggravate one of the main hallmarks of AD. Increased Aβ deposition may be linked to electrophysiological changes in irradiated TG mice as their parameters co-varied with time and radiation dose. Although our data cannot determine whether irradiation accelerates the onset of AD, they suggest that subjects with a genetic propensity to AD may exhibit increased amyloidosis as the result of exposure to low doses of proton radiation. Thus, if astronauts develop AD-related neurodegeneration in later life, their brains might exhibit increased amyloidosis due to prior exposure to space radiation. Our data further suggest that independent of genotype, exposure to protons may pathologically enhance synaptic excitability in the hippocampus and increases the risk of developing behavioral decrements.

The functional responses to proton irradiation are complex but do not appear to be synergistic with AD pathology. The absence of radiation-induced behavioral decrements in TG mice indicates that electrophysiological alterations in the hippocampus (e.g., altered neuronal firing and presynaptic glutamate release) either do not significantly contribute to their spatial learning deficits, or they develop over prolonged periods of time beyond the time scope of our study. Although the radiation-induced electrophysiological alterations appear to be insufficient to affect the AD-related behavior in mice, we speculate that similar alterations in human AD may affect clinical manifestations of the disease. Notably, the electrophysiological changes recorded in mouse hippocampus are likely taking place in other brain regions as well. In humans with much higher complexity of the CNS, changes in excitatory synaptic transmission in multiple brain regions are more likely to interfere with brain functions and modify the typical neurological manifestations of AD.

## Supporting information

S1 Fig Effects of proton radiation on survival of APP/PSEN1 TG mice up to 9 months post-irradiation. Irradiation with protons at doses up to 1 Gy did not significantly affect mortality/survival. All survival data beyond the 9-month time point are censored since the animals were sacrificed for electrophysiology. The survival curves are not significantly different between radiation groups. Statistical analyses: log-rank (Mantel-Cox test; *p* = 0.395). Mortality of WT mice is not indicated.
(TIF)

S2 Fig BM performance of the APP/PSEN1 TG and WT mice 3 and 6 months post-irradiation. (A) Performance on each of the 5 blocks. (B) Performance averaged over the blocks. Genotype effects were observed on all three tests (see analyses below). No significant radiation-induced changes were observed across time-points on any test for the TG mice. Data represent mean ± SEM. Statistical analyses of the genotype effect:
3 month post-rad., TG vs WT, Spatial 2, 2-way ANOVA: *F*_1,21_ = 9.52, †† *p* = 0.003.
6 month post-rad., TG vs WT, Cued, 2-way ANOVA: *F*_1,68_ = 12.33, ††† *p*<0.001.
6 month post-rad., TG vs WT, Spatial 1, ANCOVA: *F*_1,68_ = 3.33, *p* = 0.072.
6 month post-rad, TG vs WT, Spatial 2, ANCOVA: *F*_1,68_ = 2.59, *p* = 0.112.
(TIF)

S3 Fig Full profile I-O curves in slices from APP/PSEN1 TG and WT mice 9 months post-irradiation. (A) Presynaptic fiber volley (pV) amplitudes: Radiation exposure did not significantly affect pV amplitudes either in TG or in WT mice. Presynaptic excitability was significantly elevated in TG controls when compared to WT controls. Inset: Original trace and measurements of pV amplitude is indicated with arrows. (B) Slopes of the field excitatory postsynaptic potentials (fEPSP): Postsynaptic excitability in slices from WT mice was increased by 0.5 Gy proton irradiation. At equal dose of 0.5 Gy the fEPSP slopes were not affected in TG mice indicating qualitatively different radiation response from WT († *p* = 0.05). Inset: Original trace of the fEPSP recorded at maximal stimulation intensity in the dendritic layer of CA1 neurons. Measurement of the slope is indicated with dashed line. (C) Synaptic efficacy: The synaptic efficacy in WT mice was increased by irradiation at 0.5 Gy. In TG mice the synaptic efficacy was not affected by the exposure, but the difference between TG and WT groups at equal dose of 0.5 Gy was statistically significant. Inset: Original voltage trace indicating measurement of pV and fEPSP to compute synaptic efficacy. (D) Amplitudes of population spikes (PS): Postsynaptic spiking was significantly increased in slices from TG control (non-irradiated) mice that was visible at the initial portion of the I-O curve (at SI<1 mA); however, at higher SI the maximal PS amplitudes in TG vs WT controls were not significantly different. Irradiation at 0.1 and 1 Gy (but not at 0.5 Gy) significantly reduced the maximal amplitudes of PSs in TG mice indicating reduced hippocampal output from CA1 neurons. Inset: Original voltage trace recorded from somatic layer of CA1 neurons and indication of PS amplitude measurement. Statistical analyses in all tests: Linear Mixed Models (LMM) * *p<*0.05 TG vs TG irradiated; † *p*<0.05 WT vs TG. Data represent means ± SEM.
(TIF)

S4 Fig LTP in CA1 and CA3 neurons in slices from APP/PSEN1 TG and WT mice 6 and 9 months post-irradiation. LTP of the fEPSP was induced by high frequency (100 Hz; 2 trains 20 s apart) in CA1 and CA3 neurons by presynaptic stimulation (at time “0”) of Schaffer collaterals or Mossy fibers, respectively. LTP time course was not significantly affected by the irradiation either in TG or in WT mice. For clarity, direct comparisons of LTP recorded at 6 vs 9 months post-irradiation in TG mice are not graphed. Data represent means ± SEM.
(TIF)
