# Supplementary figures and images for: Low-dose proton radiation effects in a transgenic mouse model of Alzheimer’s disease – Implications for space travel
Source: PLoS One. 2017 Nov 29;12(11):e0186168. doi: 10.1371/journal.pone.0186168 (PMC5706673; doi:10.1371/journal.pone.0186168)

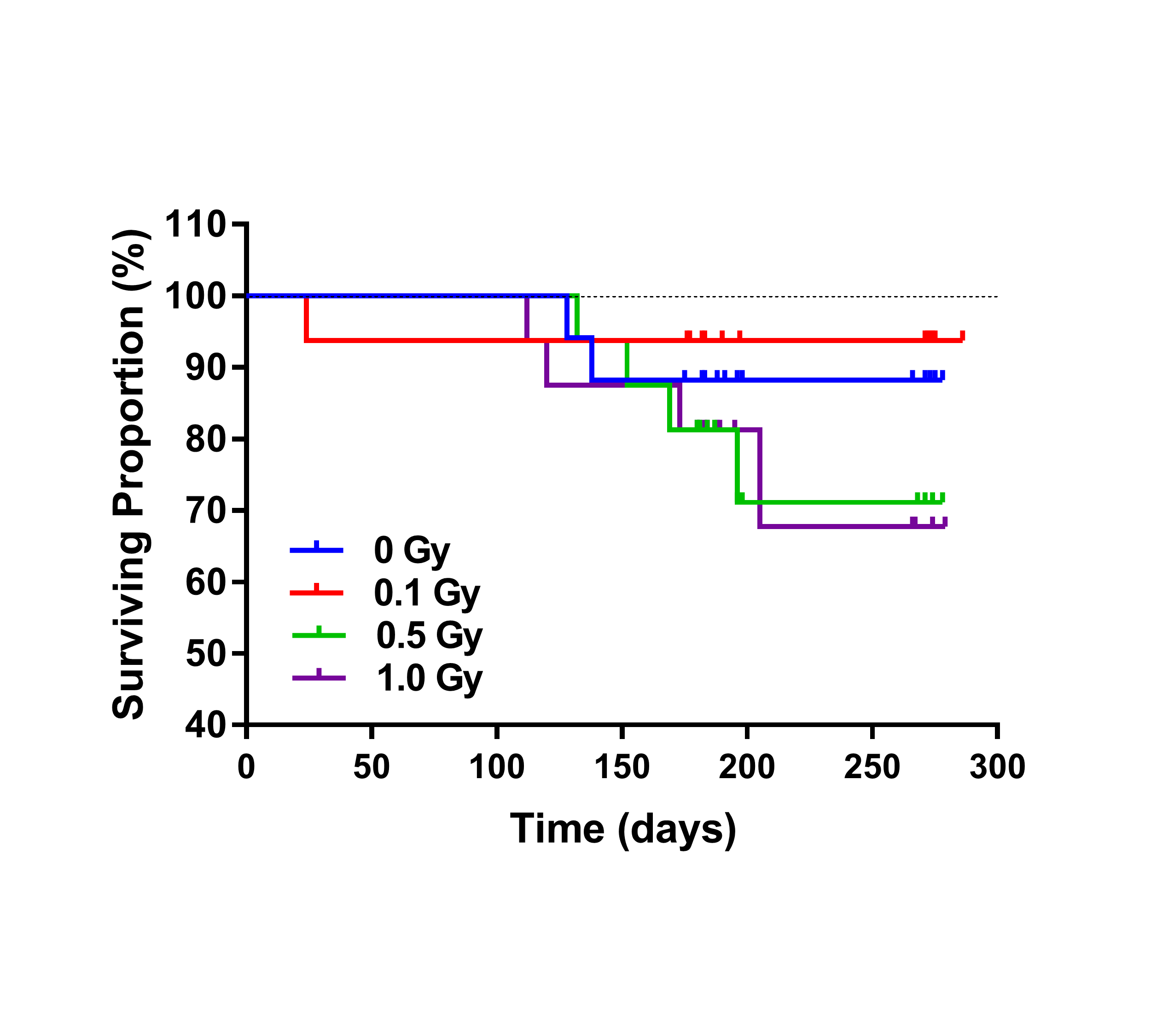

Supplement: S1 Fig — Irradiation with protons at doses up to 1 Gy did not significantly affect mortality/survival. All survival data beyond the 9-month time point are censored since the animals were sacrificed for electrophysiology. The survival curves are not significantly different between radiation groups. Statistical analyses: log-rank (Mantel-Cox test; p = 0.395). Mortality of WT mice is not indicated. (TIF) [file pone.0186168.s001.tif]

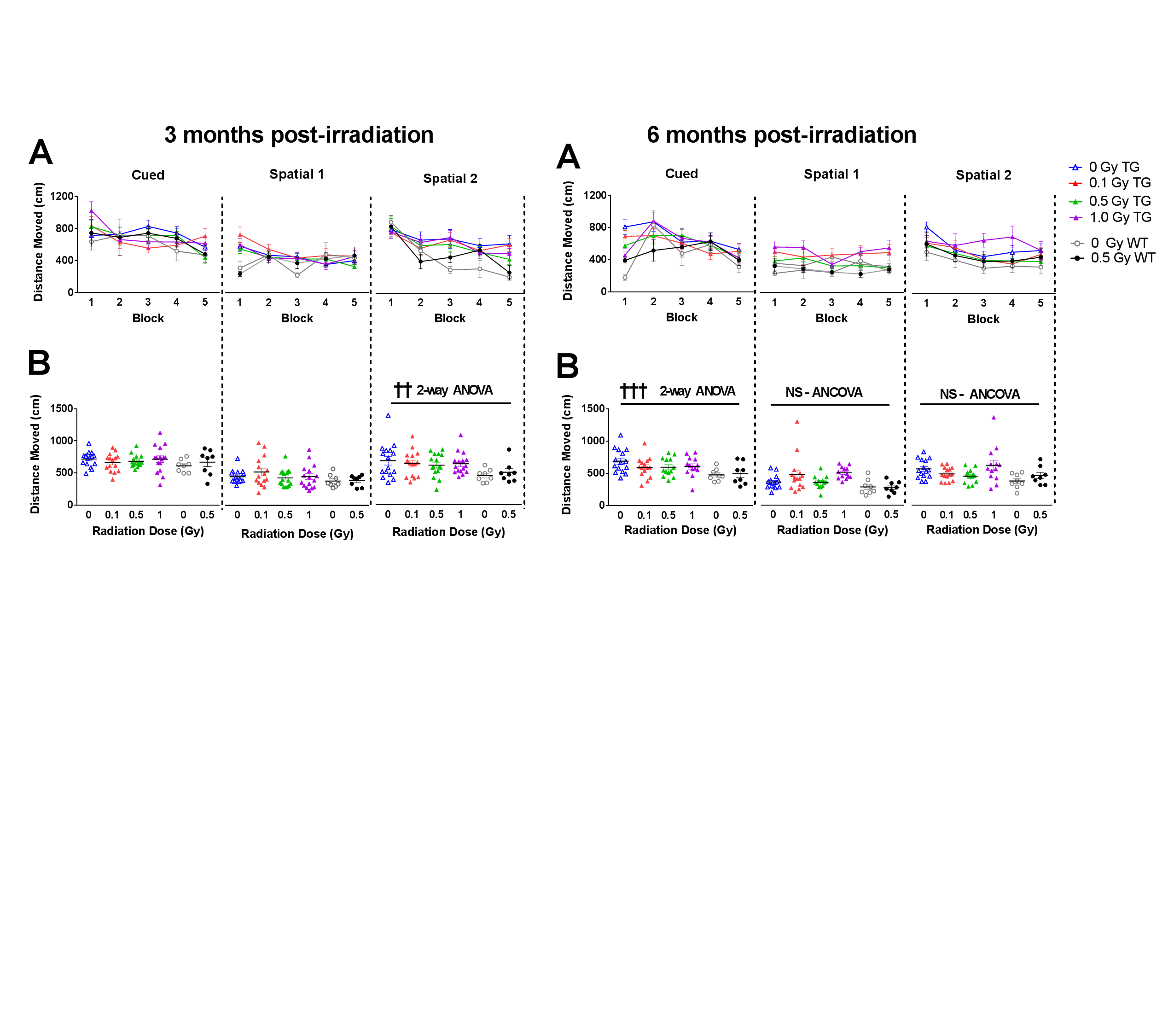

Supplement: S2 Fig — (A) Performance on each of the 5 blocks. (B) Performance averaged over the blocks. Genotype effects were observed on all three tests (see analyses below). No significant radiation-induced changes were observed across time-points on any test for the TG mice. Data represent mean ± SEM. Statistical analyses of the genotype effect: 3 month post-rad., TG vs WT, Spatial 2, 2-way ANOVA: F1,21 = 9.52, †† p = 0.003. 6 month post-rad., TG vs WT, Cued, 2-way ANOVA: F1,68 = 12.33, ††† p<0.001. 6 month post-rad., TG vs WT, Spatial 1, ANCOVA: F1,68 = 3.33, p = 0.072. 6 month post-rad, TG vs WT, Spatial 2, ANCOVA: F1,68 = 2.59, p = 0.112. (TIF) [file pone.0186168.s002.tif]

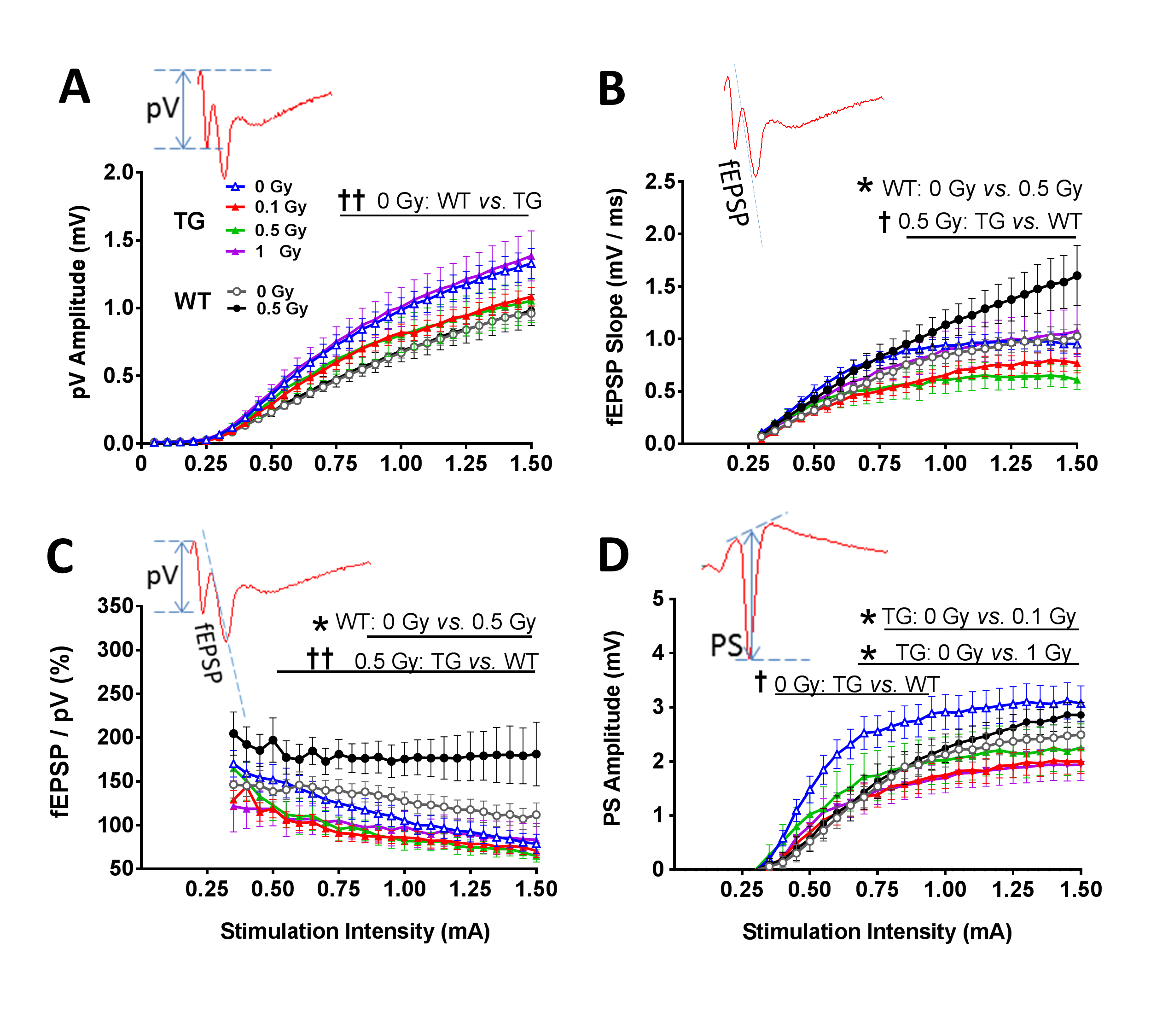

Supplement: S3 Fig — (A) Presynaptic fiber volley (pV) amplitudes: Radiation exposure did not significantly affect pV amplitudes either in TG or in WT mice. Presynaptic excitability was significantly elevated in TG controls when compared to WT controls. Inset: Original trace and measurements of pV amplitude is indicated with arrows. (B) Slopes of the field excitatory postsynaptic potentials (fEPSP): Postsynaptic excitability in slices from WT mice was increased by 0.5 Gy proton irradiation. At equal dose of 0.5 Gy the fEPSP slopes were not affected in TG mice indicating qualitatively different radiation response from WT († p = 0.05). Inset: Original trace of the fEPSP recorded at maximal stimulation intensity in the dendritic layer of CA1 neurons. Measurement of the slope is indicated with dashed line. (C) Synaptic efficacy: The synaptic efficacy in WT mice was increased by irradiation at 0.5 Gy. In TG mice the synaptic efficacy was not affected by the exposure, but the difference between TG and WT groups at equal dose of 0.5 Gy was statistically significant. Inset: Original voltage trace indicating measurement of pV and fEPSP to compute synaptic efficacy. (D) Amplitudes of population spikes (PS): Postsynaptic spiking was significantly increased in slices from TG control (non-irradiated) mice that was visible at the initial portion of the I-O curve (at SI<1 mA); however, at higher SI the maximal PS amplitudes in TG vs WT controls were not significantly different. Irradiation at 0.1 and 1 Gy (but not at 0.5 Gy) significantly reduced the maximal amplitudes of PSs in TG mice indicating reduced hippocampal output from CA1 neurons. Inset: Original voltage trace recorded from somatic layer of CA1 neurons and indication of PS amplitude measurement. Statistical analyses in all tests: Linear Mixed Models (LMM) * p<0.05 TG vs TG irradiated; † p<0.05 WT vs TG. Data represent means ± SEM. (TIF) [file pone.0186168.s003.tif]

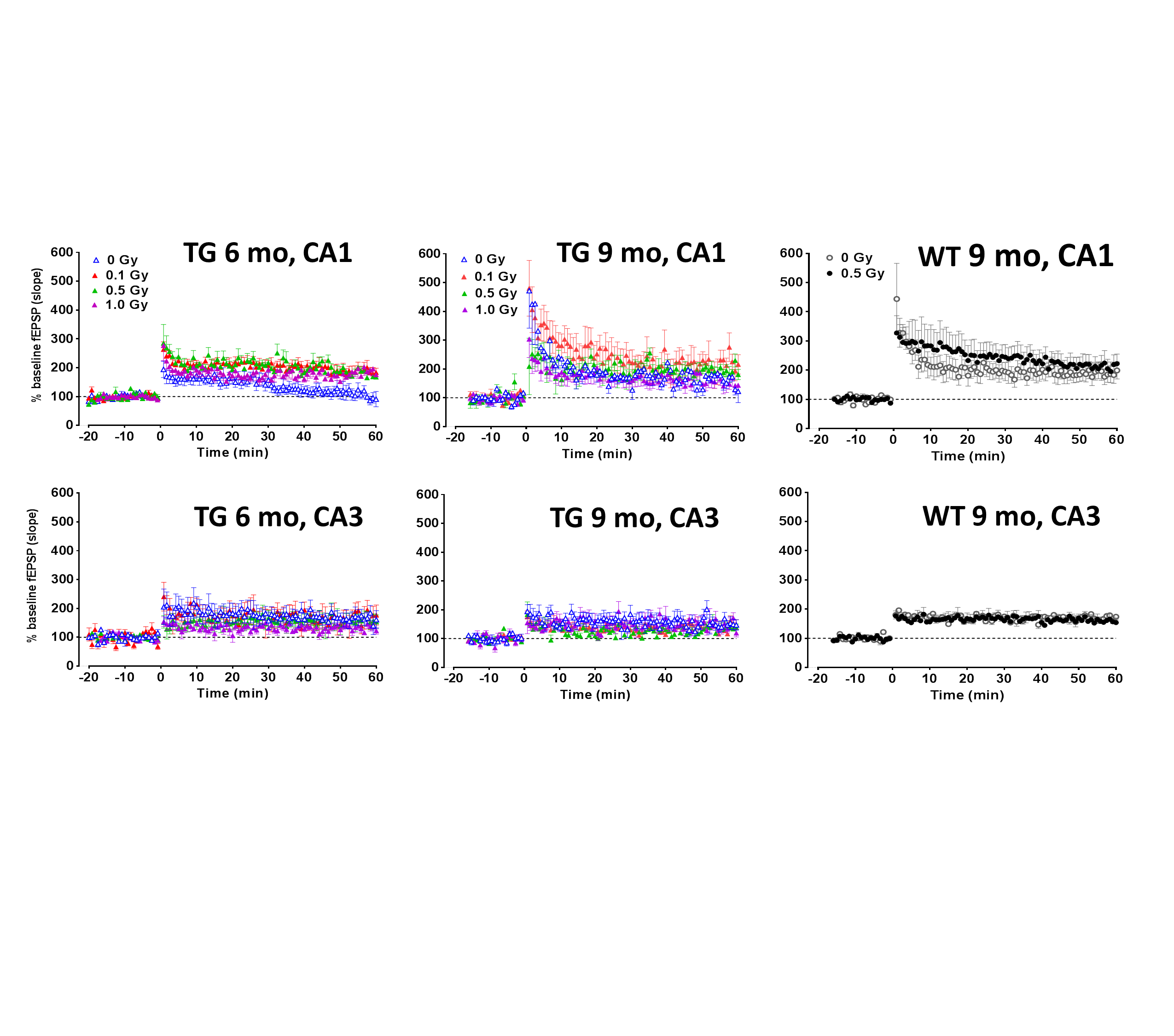

Supplement: S4 Fig — LTP of the fEPSP was induced by high frequency (100 Hz; 2 trains 20 s apart) in CA1 and CA3 neurons by presynaptic stimulation (at time “0”) of Schaffer collaterals or Mossy fibers, respectively. LTP time course was not significantly affected by the irradiation either in TG or in WT mice. For clarity, direct comparisons of LTP recorded at 6 vs 9 months post-irradiation in TG mice are not graphed. Data represent means ± SEM. (TIF) [file pone.0186168.s004.tif]
